# An RNA Sequencing Transcriptome Analysis of Grasspea (*Lathyrus sativus* L.) and Development of SSR and KASP Markers

**DOI:** 10.3389/fpls.2017.01873

**Published:** 2017-10-31

**Authors:** Xiaopeng Hao, Tao Yang, Rong Liu, Jinguo Hu, Yang Yao, Marina Burlyaeva, Yan Wang, Guixing Ren, Hongyan Zhang, Dong Wang, Jianwu Chang, Xuxiao Zong

**Affiliations:** ^1^Key Laboratory of Crop Gene Resources and Germplasm Enhancement on Loess Plateau, Ministry of Agriculture, Shanxi Key Laboratory of Genetic Resources and Genetic Improvement of Minor Crops, Institute of Crop Germplasm Resources, Shanxi Academy of Agricultural Sciences, Taiyuan, China; ^2^National Key Facility for Crop Gene Resources and Genetic Improvement, Institute of Crop Sciences, Chinese Academy of Agricultural Sciences, Beijing, China; ^3^USDA-ARS Western Regional Plant Introduction Station, Pullman, WA, United States; ^4^Department of Leguminous Crops Genetic Resources, N.I.Vavilov All-Russian Institute of Plant Genetic Resources, St. Petersburg, Russia

**Keywords:** RNA-Seq, *Lathyrus sativus*, grasspea, SSR, SNP, KASP, genetic diversity

## Abstract

Grasspea (*Lathyrus sativus* L., 2n = 14) has great agronomic potential because of its ability to survive under extreme conditions, such as drought and flood. However, this legume is less investigated because of its sparse genomic resources and very slow breeding process. In this study, 570 million quality-filtered and trimmed cDNA sequence reads with total length of over 82 billion bp were obtained using the Illumina NextSeq^TM^ 500 platform. Approximately two million contigs and 142,053 transcripts were assembled from our RNA-Seq data, which resulted in 27,431 unigenes with an average length of 1,250 bp and maximum length of 48,515 bp. The unigenes were of high-quality. For example, the stay-green (SGR) gene of grasspea was aligned with the SGR gene of pea with high similarity. Among these unigenes, 3,204 EST-SSR primers were designed, 284 of which were randomly chosen for validation. Of these validated unigenes, 87 (30.6%) EST-SSR primers produced polymorphic amplicons among 43 grasspea accessions selected from different geographical locations. Meanwhile, 146,406 SNPs were screened and 50 SNP loci were randomly chosen for the kompetitive allele-specific PCR (KASP) validation. Over 80% (42) SNP loci were successfully transformed to KASP markers. Comparison of the dendrograms according to the SSR and KASP markers showed that the different marker systems are partially consistent with the dendrogram constructed in our study.

## Introduction

Grasspea (*Lathyrus sativus* L.) is a very promising cool-season annual legume crop in many parts of the world. This plant can tolerate abiotic stress, such as drought, salinity, and flood (Kumar et al., 2011; Jiang et al., 2013; Piwowarczyk et al., 2016; Zhou et al., 2016). Grasspea also plays an important role in many low-input farming systems (Patto et al., 2006). However, this plant has several disadvantageous traits, such as containing a neurotoxin [i.e., β-N-oxalyl-L-α, β-diaminopropionic acid (β-ODAP)], indeterminate and prostrate growth habit, delayed maturation, and pod shattering (Rybinski, 2003; Yan et al., 2006; Enneking, 2011). These drawbacks impede large-scale grasspea production.

These undesirable traits of grasspea can be improved with suitable breeding strategies. For example, donor germplasm with the desirable phenotypes can be used to form new breeding materials, and available genomic tools can be applied to expedite the breeding process. However, genomic resources for grasspea are still scarce compared with other food legume crops, because grasspea has a big genome size of 8.2 Gb (Bennett and Leitch, 2012). The reference genome sequence for grasspea is unlikely to be available in the near future. Next-generation sequencing (NGS) technologies have been applied for transcriptome characterization, which is a cost-effective tool to enrich the knowledge in the genomics of grasspea. Almeida et al. (2014) generated the first comprehensive transcriptome assemblies from control and *Uromycespisi*-inoculated leaves of a susceptible and a partially rust-resistant grasspea genotype by RNA-Seq (Almeida et al., 2014).

The study of grasspea sequencing based on NGS technologies and marker development is limited by low investment and scarcity of related reports. Yang et al. (2014) developed 50,144 non-redundant SSR primers, of which 288 were randomly selected for validation among 23 *L. sativus* and one *Lathyrus cicera* accessions for diversity analysis. Among the 288 markers, 74 (25.7%) were polymorphic, 70 (24.3%) were monomorphic, and 144 (50.0%) did not amplify any PCR product (Yang et al., 2014). Almeida et al. (2014) used RNA-Seq technology to develop 200 EST-SSR markers. Among these markers, 40 markers were validated with 25 (62.5%) polymorphic between two accessions, 6 (15.0%) monomorphic, 5 (12.5%) produced a very complex pattern and the remaining 4 (10.0%) with no PCR product. Furthermore, they identified 2,634 contigs containing SNP (Almeida et al., 2014).

Kompetitive allele specific PCR (KASP) genotyping assays are based on competitive allele-specific PCR and enable bi-allelic scoring of SNPs and insertions and deletions at specific loci. This flexible and cost-effective genotyping platform was developed by LGC Limited (Fleury and Whitford, 2014; Michael, 2014; Semagn et al., 2014). KASP assays have been used in maize (Mammadov et al., 2012), wheat (Neelam et al., 2013) and peanut (Khera et al., 2013). To our knowledge, the development of KASP markers for grasspea has not been reported yet.

In this paper, we chose two different grasspea accessions and sequenced a mixture of root, stem and leaf DNA collected at the seedling stage by RNA-Seq to supply the reference of transcriptome information of grasspea. At the same time, we developed some SSR and SNP markers, which will be useful for molecular plant breeding in the future.

## Materials and methods

### Plant materials and RNA isolation

Two grasspea (*L. sativus*) accessions, in particular, one each from Africa (RQ23) and one from Europe (RQ36), were used. Each accession was sampled thrice as replications and labeled as RQ23-1, RQ23-2, RQ23-3, RQ36-1, RQ36-2, and RQ36-3 for RNA-Seq sequencing with an Illumina NextSeq^TM^ 500.

A set of 43 grasspea (*L. sativus*) accessions were used in the SSR and KASP marker tests. These germplasm resources originated in roughly 11 different geographical regions as follows: 5 accessions from Eastern Asia, 3 from Central Asia, 5 from Southern Asia, 1 from Western Asia, one from Eastern Europe, 4 from Central Europe, 1 from Northern Europe, 3 from Western Europe, 14 from Southern Europe, 4 from Eastern Africa and 2 from Northern Africa.

The seed samples were obtained from the Institute of Crop Germplasm Resources, Shanxi Academy of Agricultural Sciences, Taiyuan, China. Detailed information is given in Table S4.

### RNA-Seq library preparation and illumina sequencing

RNA from each of the samples, which included mixtures of root, stem and leaf in the seedling stage (3 weeks after sowing), was extracted using the RNA prepPure Plant Kit (Tiangen, Beijing, China) according to manufacturer's instructions. Oligo-dT labeled magnetic beads (Illumina Inc., San Diego, USA) were used to combine the polyA of the mRNA for purifying the mRNA. Then mRNA was mixed with fragmentation buffer to obtain short fragment RNAs with the size of 200–300 bp. Then, the short fragment RNAs were used to synthesize the first-strand cDNA with random primers, and this cDNA was transformed into double-strand cDNA using RnaseH and DNA polymerase I. Fragments of desirable lengths (200–300 bp) were purified by the QIAquick PCR Extraction Kit (Qiagen, Valencia, CA, USA). Under the function of 3′-5′ exonuclease and polymerase, the protruding termini of the DNA fragments were end-repaired. The end-repaired DNA fragments were ligated with sequencing adapters through A and T complementary base pairing. Then, AMPure XP beads (Beckman Coulter, Shanghai, China) were used to remove unsuitable fragments. The sequencing library was constructed by PCR. The multiplexed cDNA libraries were tested using PicoGreen (Quantifluor™-ST fluorometer E6090, Promega, CA, USA) and fluorospectrophotometry (Quant-iT PicoGreen dsDNA Assay Kit; Invitrogen, P7589) and quantified with Agilent 2100 (Agilent 2100 Bioanalyzer, Agilent 2100; Agilent High Sensitivity DNA Kit, Agilent, 5067–4626). Furthermore, the synthesized cDNA libraries were normalized to 10 nM. Finally, the sequencing library was gradually diluted and quantified to 4–5 pM and sequenced on the Illumina NextSeq™ 500 system. The raw data were deposited in the Sequence Read Archive (SRA) in NCBI as SRP092875.

### Data filtering and *de novo* assembly

After the sequencing of the Illumina paired-end, the raw reads were filtered by removing the adapter sequences, reads that contain unknown bases of more than 10%, and reads with a low quality score (*Q* < 20). Trinity, r20140717 (https://github.com/trinityrnaseq/trinityrnaseq/wiki) was used to assemble high-quality reads into contigs and transcripts, and the k-mer was equal to 25. Data redundancy was reduced by clustering the transcripts by blasting against the nr protein database with a cut-off e-value of 1e^−5^. Then, the longest sequences in each cluster were reserved as unigenes.

### Unigene annotation and classification

The unigenes were aligned with BLASTX to five protein databases, namely, NCBI non-redundant protein sequences (Nr) (with a cut-off e-value of 1e^−5^), Gene Ontology (GO) (using Blast2go and map2slim software), Kyoto Encyclopedia of Genes and Genome (KEGG) (using bi-directional best hit method), and evolutionary genealogy of genes: Non-supervised Orthologous Groups (eggNOG) (with a cut-off e-value of 1e^−5^) and Swiss-Prot (with a cut-off e-value of 1e^−5^).

### Aligning an annotated grasspea gene with its homologue in pea

The pea SGR mRNA sequences on NCBI were searched using keywords AB303331 and AB303332. Grasspea unigenes were transformed into a blast database by using CLC Genomics Workbench 9_0_1 software (CLC Inc., Aarhus, Denmark). Then AB303331 and AB303332 data sets were blasted against the grasspea unigene database with default parameters. The grasspea unigene with the most significant alignment score aligned with the gene, c39901_g1_il, a senescence-inducible chloroplast stay-green (SGR) protein. c39901_g1_i1 was SGR homolog gene in grasspea. Then c39901_g1_i1, AB303331 and AB303332 were translated to protein using ORF finder software on the NCBI website (https://www.ncbi.nlm.nih.gov/orffinder/). The longest translated protein was selected for further analysis. Finally, multi-aligning was finished by the ClustalW Multiple function of BioEdit version 7.3.5 (12/22/2013) (Hall, 1999).

### SSR search and primer design

High-throughput SSR search was performed by microsatellite identification tool (version 1.0, http://pgrc.ipk-gatersleben.de/misa/misa.html). The parameters were set as follows: minimum SSR motif repeat length of mono-10, di-6, tri-5, tetra-5, penta-5, and hexa-5. The maximum size of interruption allowed between two different SSRs in a compound SSR was 100 bp. SSR primer pairs were designed based on flanking conserved sequences and the microsatellite loci were selected using the Primer 3.0 (https://sourceforge.net/projects/primer3/) (Rozen and Skaletsky, 2000).

### DNA extraction and PCR amplification

Genomic DNA was extracted from the fresh leaves of seedlings (3 weeks after sowing) of 43 accessions using the Rapid Plant Genomic DNA Isolation Kit (B518231-0100, SangonBioteck, Shanghai, China) and the laboratory procedures were conducted strictly according to the manufacturer's instructions. DNA qualities were tested by the BioTek Synergy H1 and the PCR concentration of DNA was diluted to 30 ng/μL to confirm the markers. Amplification reaction system was as follows: 10 μL volume containing 0.1 μL of Taq DNA polymerase (5 U/μL, Aidlab, Beijing, China), 2 μL of primers (12 ng/μL, Personalbio, Shanghai, China), 1 μL 10 × buffers (Aidlab, Beijing, China), 0.25 μL of dNTP (10 mM, SangonBioteck, Shanghai, China), 5.15 μL of ultrapure water (Millipore Direct-Q3), and 1.5 μL of genomic DNA (30 ng/μL). Microsatellite loci were amplified on the C1000 Thermal Cycler (Bio-rad, USA). PCR amplification was performed under the following cycling conditions: primary of one cycle for 5 min at 95°C; 35 cycles at 95°C for 30 s, 52°C for 45 s, and 72°C for 45 s; and final extension at 72°C for 10 min. The PCR products were tested by 6.0–8.0% non-denaturing Polyacrylamide gel electrophoresis using silver nitrate staining for visualization.

### SSR marker polymorphic validation

The parameters of genetic diversity were determined by calculating the screening data of SSR markers, using PowerMarker (Version 3.25) (http://statgen.ncsu.edu/powermarker/). These parameters included the major allele frequency, number of alleles, gene diversity (GD), heterozygosity, and polymorphic information content (PIC) in SSR polymorphic markers.

### SNP search

The Bowtie2 (version 2.2.4) software was used to map the high-quality reads to unigenes according to the default parameter. Then, Samtools (version 1.1) (Li et al., 2009) was used to generate bam files. Varscan (version 2.3.7) (Koboldt et al., 2012) was used to call SNP according to the parameter as follows: mincoverage [8], min reads [2], min varfreq [0.2], min avgqual [15], *p*-value [0.01].

### KASP primer design and validation

KASP primers were designed according to the standard KASP guidelines. The allele-specific primers were designed carrying the FAM (5′–GAAGGTGACCAAGTTCATGCT-3′) and HEX (5′-GAA-GGTCGGAGTCAACGGATT-3′) tails with the targeted SNP at the 3′ end. 1,536-well plates were used to genotype each sample with 1 μL of reaction mix as follows: dry DNA, 0.5 μL of 2× Master mix, 0.014 μL of Primer mix, and 0.486 μL of ddH_2_O. All reagents were briefly vortex-mixed prior to use. The KASP thermal cycling program was as follows: 94°C for 15 min; then 10 cycles at 94°C for 20 s, 61–55°C for 60 s (decrement of 0.6°C per cycle); and 26 cycles at 94°C for 20 s and 55°C for 60 s. Fluorophores FAM and HEX were used to distinguish genotypes. Snpviewer2 was used to view the result of KASP markers. Major allele frequency, number of alleles, GD, heterozygosity and PIC in the SNP polymorphic markers were calculated and these indices were the same as those in the validation of SSR markers.

### Construction of phylogenetic dendrograms

Phylogenetic dendrograms were constructed based on the screening data of SSR and SNP markers. PowerMarker (version 3.25) was used to calculate the frequency and genetic distance (Nei and Roychoudhury, 1974) and build the phylogenetic original tree and bootstrap consensus tree by Unweighted pair-group method with arithmetic means (UPGMA), which was based on bootstrap 1,000 times. Eventually, the dendrograms were drawn by MEGA (version 5.1) (http://www.megasoftware.net/download_form).

## Results

### Illumina sequencing and *de novo* assembly of the grasspea

A total of 111.8, 86.9, 107.7, 90.0, 84.3, and 99.1 million raw reads were generated by the Illumina NextSeq™ 500 system for RQ23-1, RQ23-2, RQ23-3, RQ36-1, RQ36-2, and RQ36-3, respectively. After removal of the adaptor and low quality reads, approximately 109.7, 85.2, 106.1, 88.1, 83.2, and 98.0 million clean reads remained for RQ23-1, RQ23-2, RQ23-3, RQ36-1, RQ36-2, and RQ36-3, respectively. The combined sequences of these clean reads were assembled into 142,053 transcripts and 27,431 unigenes. Table 1 shows that the N50 of the transcript was 1,294 bp and the average length was 846 bp. Meanwhile, the N50 of the unigenes was 1,781 bp and the average length was 1,250 bp.

**Table 1:** Characteristics of *de novo* assembly of the grasspea by Trinity software in this study.

	**Transcript**	**Unigene**
Total Length (bp)	120,219,755	34,278,781
Sequence Number	142,053	27,431
Max. Length (bp)	48,514	48,514
Mean Length (bp)	846	1,250
N50 (bp)	1,294	1,781
N50 Sequence No.	28,623	6,486
N90 (bp)	352	637
N90 Sequence No.	97,051	18,215
GC%	39.77	40.28

### Gene annotation and functional classification

**Figure 1:**
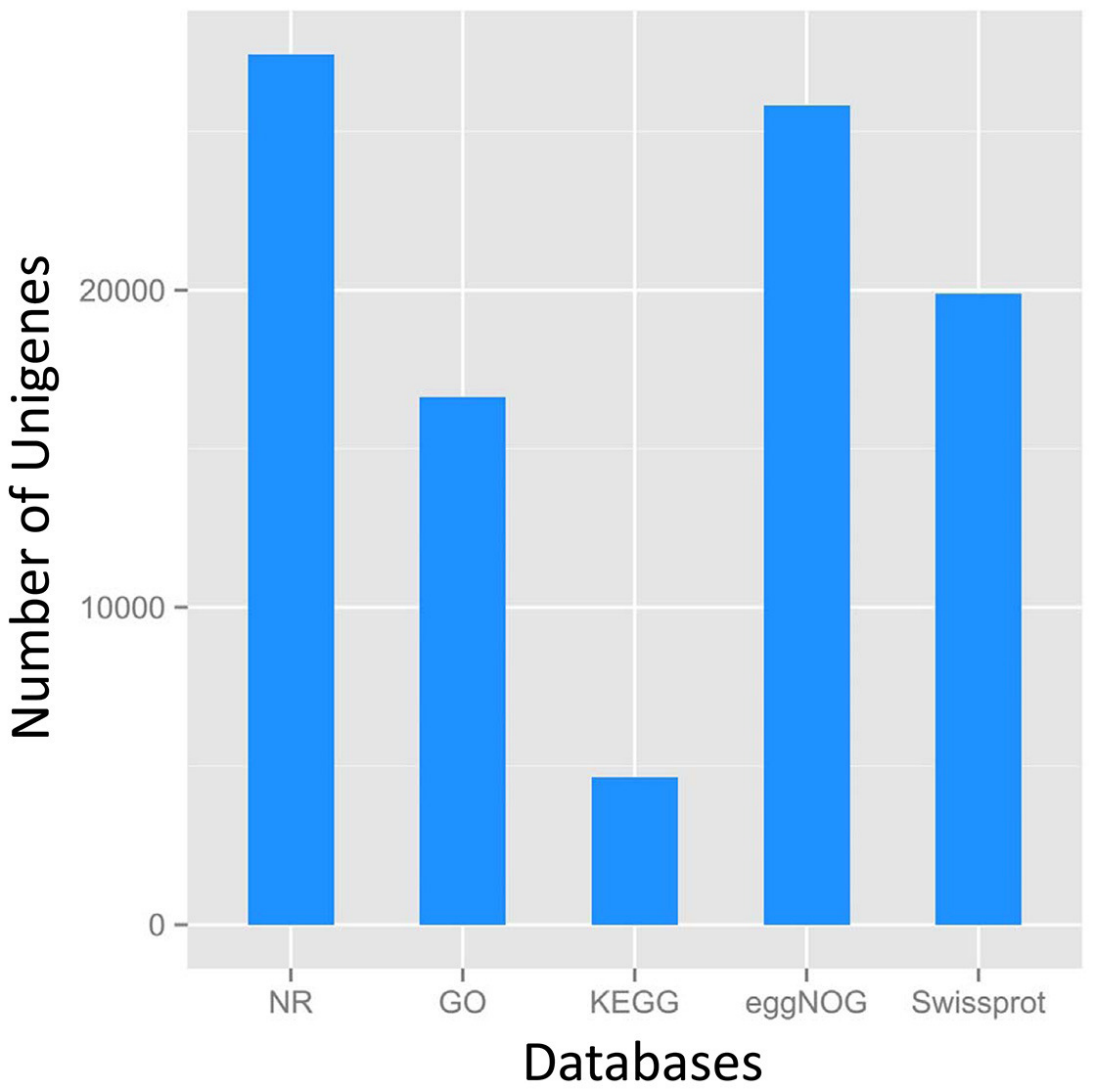
Number distribution of annotation results of Unigenes in Nr, GO, KEGG, eggNOG, Swissport database.

**Figure 2:**
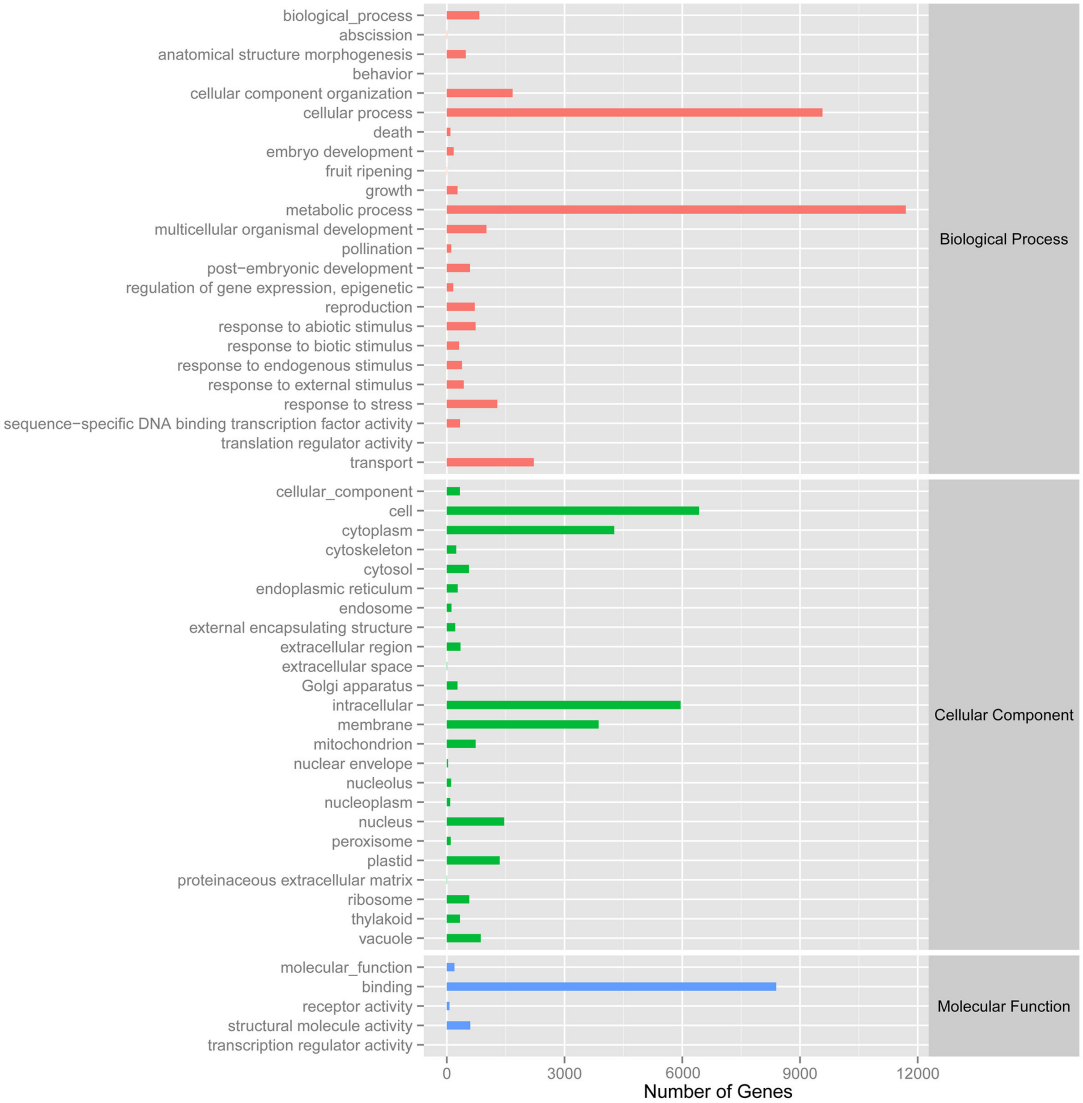
Number distribution of GOSlim annotation of Unigenes related to biological process, cellular component and molecular function.

A total of 27,431 unigenes provided a significant BLASTX result with 27,431 (100%) showing significant similarity to NCBI non-redundant (Nr) protein sequences and 19,867 (72.4%) from Swiss-Prot (Figure 1). The transcriptome of grasspea was functionally annotated using BLAST2GO according to the default parameter (Conesa et al., 2005; Götz et al., 2008). Map2slim script mapped the gene association file (containing annotations to the full GO) to the terms in the GO slim. Figure 2 shows that metabolic process was the most frequent category in biological processes, the cell was the most frequent category in cellular component, and binding was the most frequent category in molecular function.

**Figure 3:**
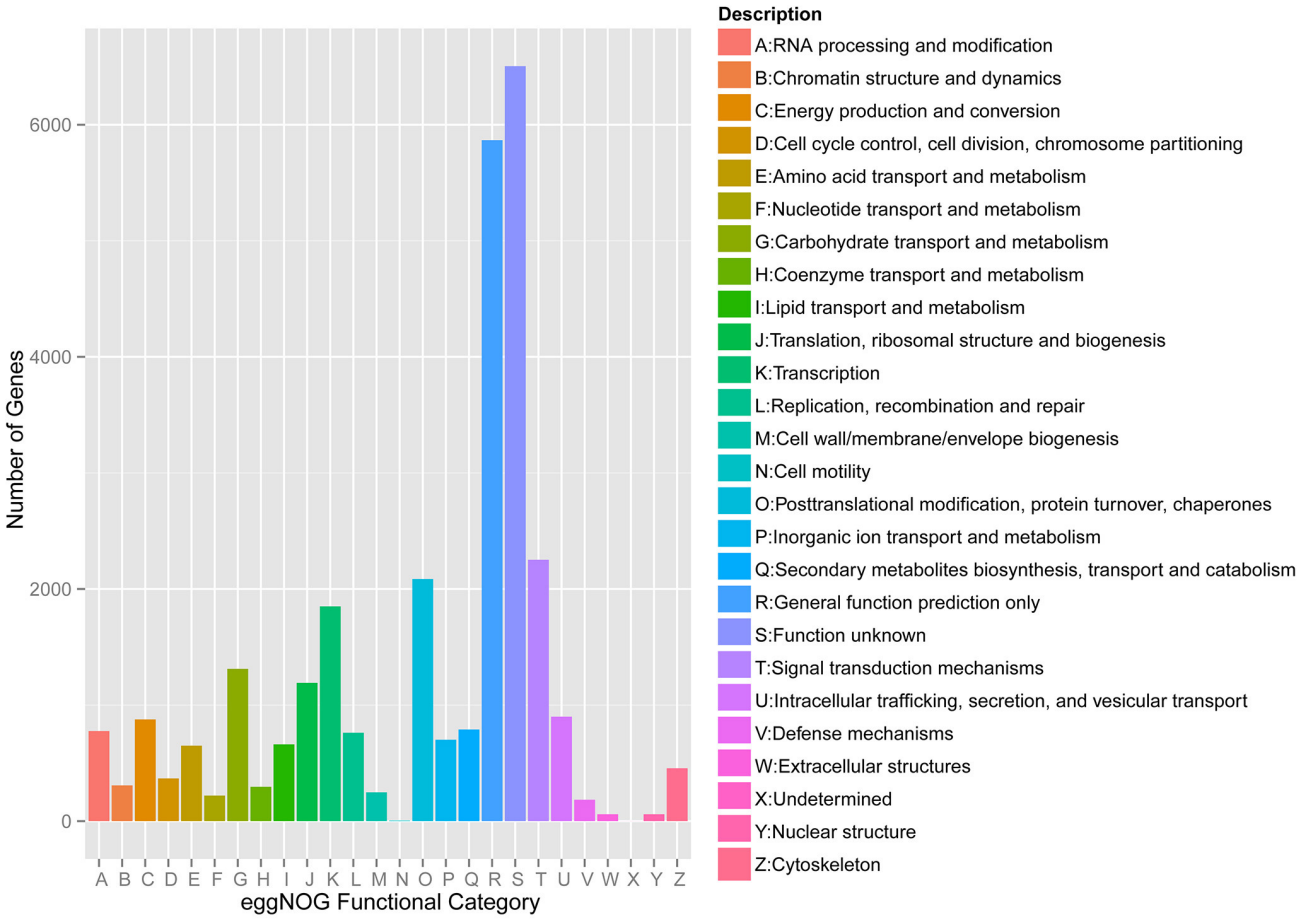
Number distribution of eggNOG annotation of Unigenes related to A-Z.

**Figure 4:**
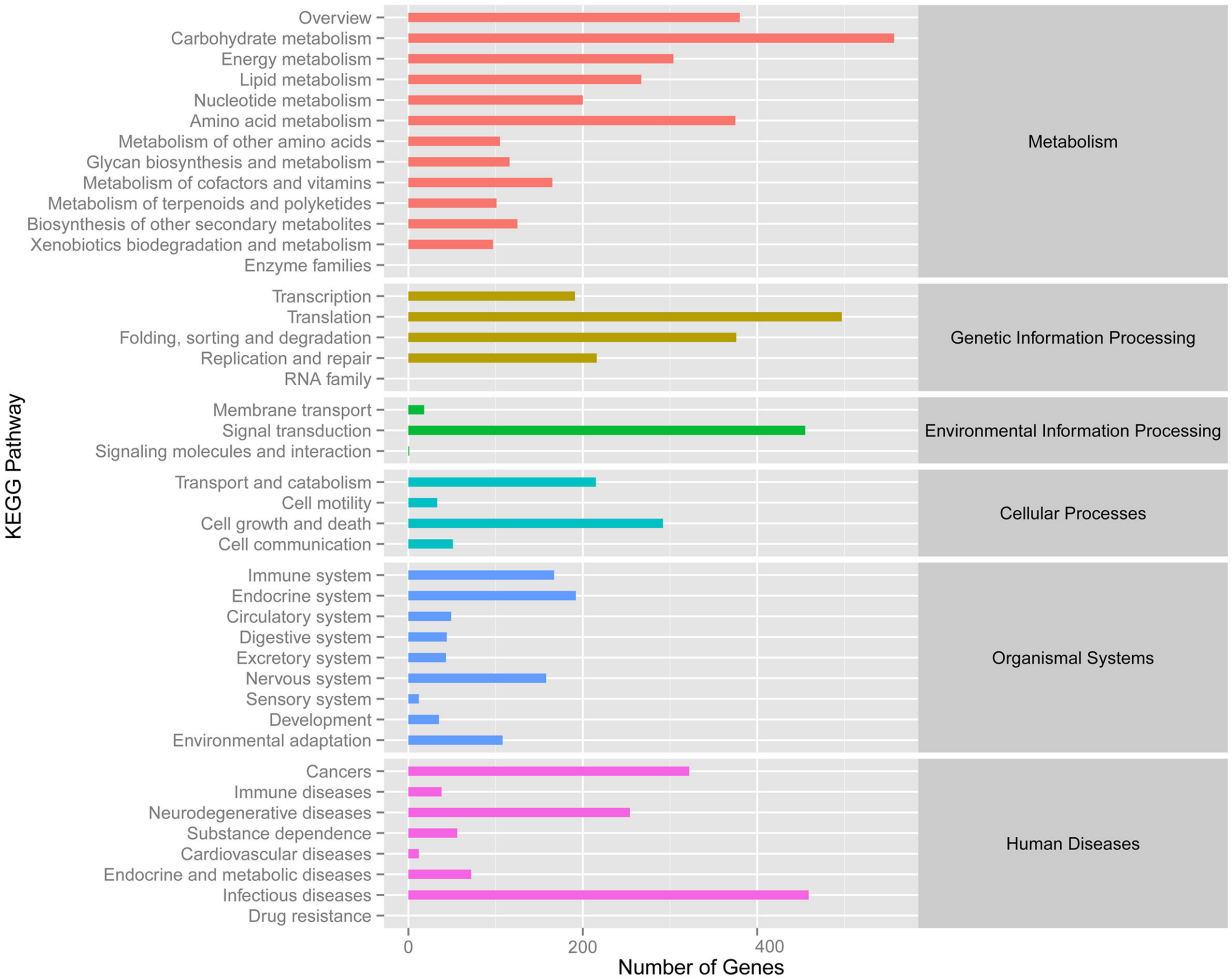
Number distribution of KEGG annotation of Unigenes related to metabolism, genetic information processing, environmental information processing, cellular processes, organismal systems and human diseases.

**Figure 5:**
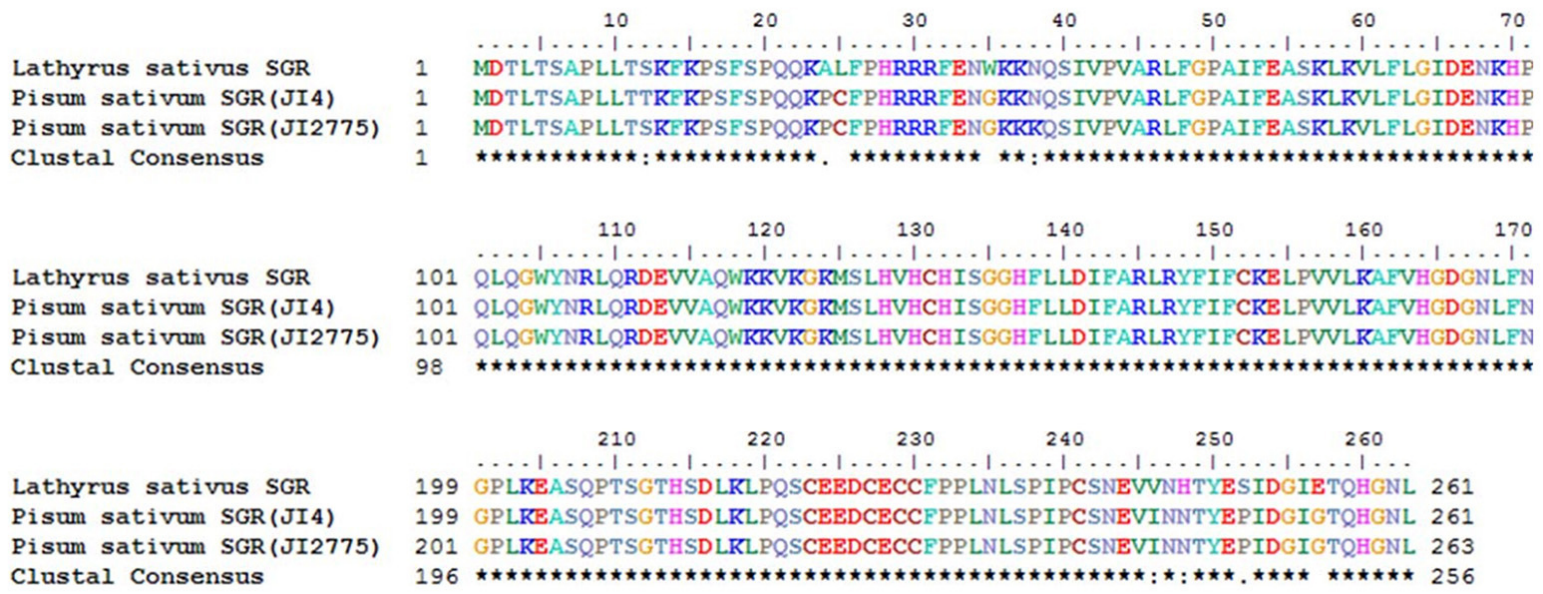
Alignment between unigene c39901_g1_i1 annotated for senescence-inducible chloroplast stay-green protein of grasspea and SGR gene of pea.

Meanwhile, eggNOG annotation was finished by blasting against the eggNOG (Version 4.0) database. A total of 25,822 unigenes were annotated. Figure 3 shows that unknown function and general function prediction were the most frequent categories. Undetermined and cell motility were the least frequent categories. KEGG pathway was analyzed in our study. Figure 4 shows that carbohydrate metabolism, transcription, signal transduction, cell growth and death, endocrine system and infectious diseases were the most frequent categories in metabolism, followed by genetic information processing, environmental information processing, cell processes, organismal systems and human diseases. Interestingly, c39901_g1_i1 annotated for senescence-inducible chloroplast SGR protein was the SGR gene in grasspea. Sato et al. (2007) found that pea SGR was Mendel's green cotyledon gene (*I/i*) encoding a positive regulator of the chlorophyll-degrading pathway in pea. Figure 5 illustrates that the SGR gene of grasspea was aligned with the SGR gene of pea with high similarity (Sato et al., 2007; Hradilová et al., 2017).

### Polymorphic validation of EST-SSR markers

A total of 3,204 EST-SSR primers were designed (Table S2) and 284 (Table S3) were randomly selected for validation. The EST-SSR markers were validated with 43 grasspea accessions mentioned previously. The result showed that 87 polymorphic and 88 monomorphic markers were confirmed, which accounted for the 30.6 and 31.0% of 284 markers, respectively.

Table 2 shows that the number of alleles was from 2 to 8 with a mean value of 3.6. The PIC values varied from 0.0848 to 0.7425 with mean value of 0.4158. These results, suggested that these EST-SSRs were informative markers and useful for marker-assisted breeding in the future.

**Table 2:** Results of 87 effective primers screening in 43 accessions of *Lathyrus sativus* L.

**No**.	**Marker**	**Allele no**.	**GeneDiversity**	**Heterozygosity**	**PIC**
1	9	6	0.7633	0.0952	0.7312
2	10	4	0.3818	0.0000	0.3473
3	11	7	0.7434	0.1628	0.7043
4	13	6	0.5488	0.0000	0.5167
5	17	2	0.1687	0.0000	0.1545
6	18	3	0.5581	0.1628	0.4748
7	19	5	0.3883	0.0000	0.3614
8	22	3	0.5105	0.3256	0.4195
9	24	5	0.3772	0.0233	0.3579
10	27	4	0.5054	0.0233	0.4260
11	32	3	0.5365	0.0000	0.4342
12	34	3	0.5498	0.1395	0.4498
13	37	2	0.1874	0.0698	0.1698
14	38	3	0.3743	0.0000	0.3308
15	41	2	0.4543	0.0000	0.3511
16	42	3	0.5744	0.0465	0.5113
17	44	5	0.5027	0.0698	0.4513
18	46	3	0.6224	0.0000	0.5436
19	48	3	0.3350	0.1163	0.3076
20	49	5	0.4075	0.1395	0.3831
21	50	4	0.3256	0.0000	0.3097
22	51	6	0.6666	0.1163	0.6378
23	58	5	0.6533	0.0238	0.5923
24	61	3	0.3916	0.0000	0.3310
25	62	4	0.3748	0.0000	0.3350
26	63	2	0.4867	0.0000	0.3683
27	64	3	0.5430	0.0930	0.4741
28	65	3	0.5127	0.0000	0.4030
29	66	3	0.5525	0.2326	0.4763
30	67	3	0.5222	0.0698	0.4080
31	70	3	0.5741	0.0233	0.5109
32	72	3	0.6088	0.0000	0.5390
33	73	4	0.4770	0.0930	0.4147
34	77	2	0.0887	0.0000	0.0848
35	86	3	0.5527	0.1860	0.4590
36	97	3	0.4248	0.0465	0.3522
37	100	2	0.4932	0.0000	0.3716
38	104	5	0.6721	0.3095	0.6141
39	110	4	0.5023	0.0000	0.4320
40	116	4	0.5365	0.0465	0.4904
41	120	2	0.4781	0.0000	0.3638
42	131	5	0.6844	0.1395	0.6225
43	133	3	0.4429	0.0244	0.4013
44	134	5	0.3916	0.0465	0.3685
45	143	6	0.7380	0.1628	0.6959
46	144	5	0.5628	0.1351	0.4673
47	147	6	0.7542	0.2093	0.7191
48	148	4	0.6552	0.0930	0.6076
49	155	3	0.3974	0.0000	0.3616
50	158	5	0.7785	0.1628	0.7425
51	160	4	0.3167	0.0233	0.2890
52	162	4	0.6763	0.3256	0.6161
53	165	3	0.5560	0.1860	0.4956
54	167	5	0.2718	0.0233	0.2606
55	168	3	0.3104	0.0000	0.2746
56	174	4	0.5979	0.0698	0.5523
57	179	3	0.5322	0.0000	0.4721
58	180	2	0.3029	0.0000	0.2570
59	183	3	0.5162	0.1628	0.4228
60	193	4	0.3469	0.0465	0.3120
61	195	4	0.2117	0.0465	0.2003
62	200	2	0.4770	0.2143	0.3633
63	202	3	0.3396	0.0000	0.2956
64	203	4	0.6414	0.0465	0.5776
65	205	4	0.4010	0.0465	0.3509
66	206	3	0.2258	0.1163	0.2050
67	210	4	0.6414	0.2558	0.5788
68	214	4	0.3213	0.0465	0.2997
69	221	4	0.5298	0.0238	0.4407
70	226	2	0.4082	0.0000	0.3249
71	228	2	0.3442	0.1163	0.2850
72	230	4	0.4227	0.1860	0.3963
73	231	2	0.1298	0.0000	0.1214
74	235	2	0.4082	0.0000	0.3249
75	240	3	0.5790	0.2326	0.5024
76	244	3	0.2120	0.0000	0.2010
77	250	2	0.3442	0.2093	0.2850
78	253	5	0.5484	0.0000	0.4607
79	257	5	0.6266	0.2326	0.5742
80	261	2	0.3569	0.0930	0.2932
81	269	3	0.5000	0.0000	0.4275
82	270	2	0.1298	0.0000	0.1214
83	272	4	0.2565	0.0952	0.2436
84	273	2	0.3442	0.0698	0.2850
85	279	4	0.6617	0.2326	0.6053
86	281	2	0.4543	0.1395	0.3511
87	283	8	0.7593	0.3333	0.7300

### Polymorphic validation of KASP markers

A total of 146,406 SNP (Table S4) were detected in this study and 50 SNP loci (Table S5) were randomly selected for KASP validation. Consequently, 42 SNP loci were successfully transformed to KASP markers. Two of these loci were monomorphic and the others were polymorphic among 43 accessions. Table 3 shows that the PIC values ranged from 0 to 0.3750 with an average of 0.2457. Comparative results show that the KASP markers were less informative than EST-SSR markers for the lower PIC values. Since the transform ratio from SNP to KASP markers was high, the SNP markers associated with desirable traits will be easily converted to KASP for marker-assisted selection in the future.

**Table 3:** Results of 42 KASP primers screening in 43 accessions of *Lathyrus sativus* L.

**No**.	**Marker**	**Allele No**.	**Gene Diversity**	**Heterozygosity**	**PIC**
1	c24137	2.0000	0.4730	0.1163	0.3611
2	c29065	2.0000	0.2726	0.0930	0.2354
3	c29470	2.0000	0.4640	0.1951	0.3564
4	c31876	2.0000	0.4997	0.0714	0.3749
5	c31909	2.0000	0.0454	0.0000	0.0444
6	c34057	2.0000	0.4673	0.1860	0.3581
7	c34112	2.0000	0.2401	0.1395	0.2113
8	c34138	2.0000	0.1095	0.0698	0.1035
9	c34320	2.0000	0.2566	0.0698	0.2237
10	c35446	2.0000	0.0887	0.0000	0.0848
11	c36697	2.0000	0.3691	0.2093	0.3010
12	c36972	2.0000	0.4976	0.0930	0.3738
13	c36982	2.0000	0.4024	0.0465	0.3214
14	c37578	1.0000	0.0000	0.0000	0.0000
15	c40344	2.0000	0.0887	0.0000	0.0848
16	c40733	2.0000	0.1095	0.0698	0.1035
17	c40873	2.0000	0.4308	0.1628	0.3380
18	c41137	2.0000	0.4827	0.0698	0.3662
19	c41578	2.0000	0.4308	0.2558	0.3380
20	c41707	2.0000	0.4957	0.3023	0.3728
21	c41761	2.0000	0.1298	0.0465	0.1214
22	c41833	1.0000	0.0000	0.0000	0.0000
23	c41924	2.0000	0.4673	0.1395	0.3581
24	c41959	2.0000	0.2566	0.0698	0.2237
25	c42781	2.0000	0.1095	0.0698	0.1035
26	c43192	2.0000	0.0454	0.0000	0.0444
27	c43598	2.0000	0.5000	0.2093	0.3750
28	c43850	2.0000	0.4976	0.2791	0.3738
29	c43880	2.0000	0.4730	0.0698	0.3611
30	c44108	2.0000	0.3918	0.1628	0.3151
31	c44112	2.0000	0.4673	0.1395	0.3581
32	c44175	2.0000	0.4470	0.2093	0.3471
33	c44559	2.0000	0.3569	0.1395	0.2932
34	c45088	2.0000	0.4997	0.1395	0.3749
35	c45396	2.0000	0.4611	0.2093	0.3548
36	c45432	2.0000	0.1298	0.0465	0.1214
37	c45786	2.0000	0.0230	0.0233	0.0227
38	c46069	2.0000	0.2055	0.0930	0.1844
39	c46261	2.0000	0.0673	0.0233	0.0651
40	c47018	2.0000	0.3569	0.0000	0.2932
41	c47065	2.0000	0.3691	0.0698	0.3010
42	c47339	2.0000	0.4976	0.6977	0.3738

### Comparison of dendrograms according to SSR and KASP markers

**Figure 6:**
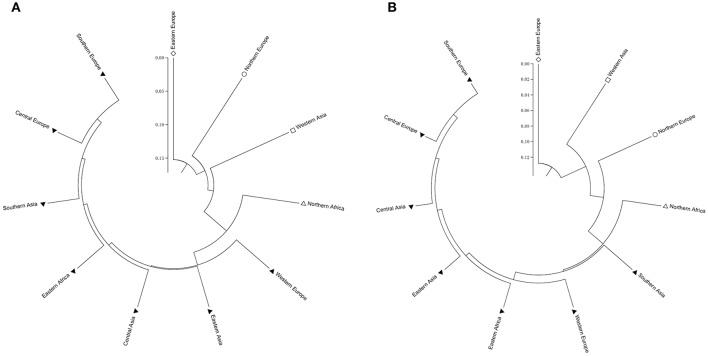
Two UPGMA dendrograms showing the relationship of the geographic groups of the 43 grasspea accessions using Nei and Roychoudhury's (1974) genetic distance. **(A)** Based on 87 SSR markers and **(B)** based on 42 SNP markers.

**Figure 7:**
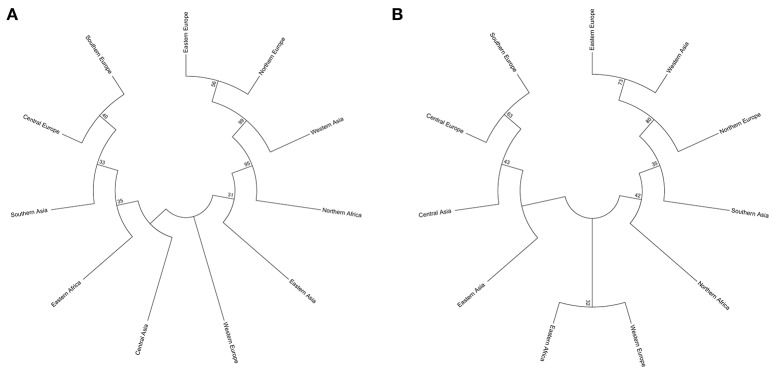
Two bootstrap consensus trees showing the relationship of the geographic groups of the 43 grasspea accessions using 1,000 bootstraps. **(A)** Based on 87 SSR markers and **(B)** based on 42 SNP markers.

Two types of UPGMA dendrograms, including an original tree and a bootstrap consensus tree were constructed based on the genotype data of 87 SSR and 40 KASP markers for the 43 accessions. The frequency was calculated using Nei's genetic distance coefficient (Nei and Roychoudhury, 1974) and bootstrapping 1,000 times. The 11 subgroups based on different geographical origin were grouped into five groups as follows: (1) Southern Europe, Central Europe, Southern Asia, Eastern Africa, Central Asia, Eastern Asia and Western Europe; (2) Northern Europe; (3) Eastern Europe; (4) Western Asia; and (5) Northern Africa (Table S1). Although minor differences exist between the two dendrograms, the relationships among subgroups are similar. The results suggest that these SSR and KASP markers are useful for assessing genetic diversity of grasspea genetic resources (Figures 6A,B). Through the bootstrap consensus analysis, bootstrap values > 30% were presented. The results showed that the accessions from Eastern Europe, Northern Europe, Western Asia and Northern Africa were in one group that was supported by bootstrap values between 70 and 95% based on SSR markers. Other accessions were weakly supported because of bootstrap values < 50%. In the terms of KASP markers, accessions from Eastern Europe, Western Asia and Northern Europe similarly fell into one group, except for Northern Africa, which showed partial consistency as well (Figures 7A,B).

## Discussion

### Grasspea is an orphan crop with great potential

Grasspea is an under-researched legume crop with a big genome. This legume, as a minor crop, is very important in the arid and semi-arid regions, such as the Mediterranean, Middle East region and Southern Asian subcontinent, in particular, Italy, Spain, Egypt, Greece, Turkey, Ethiopia, Syria, India and Bangladesh (Patto et al., 2006; Yan et al., 2006; Dixit et al., 2016). Several research groups have paid attention to grasspea and its wild relatives (*L. cicera* L.) because of the high resistance of these plants to both abiotic and biotic stresses, such as drought, flooding and saline-alkali, certain diseases and pests (Wang et al., 2015). However, grasspea is difficult to apply for large scale agricultural production worldwide because of its big genome (8.2 Gb), variable outcrossing rate (2–30%) (Rahman et al., 1995; Chowdry and Slinkard, 1997; Hillocks and Maruthi, 2012) and the presence of β-ODAP in its seed. With the help of NGS, the complex trait-related genes are easier to determine with RNA-Seq, RAD-Seq, Chip-Seq and GBS technologies (Singh and Singh, 2015). In the study, we applied RNA-Seq for two different accessions, namely, one from Africa and the other from Europe, to assemble the gene reference of grasspea. The result will be useful for gene mining and molecular breeding to improve grasspea.

### SSR markers are still effective and useful

The SSR markers are co-dominant, have abundant polymorphism and ubiquity in many eukaryotic species (Zietkiewicz et al., 1994), have high repeatability and are user-friendly. SSR is a powerful marker for germplasm evaluation and smart breeding. Numerous research groups use this molecular tool in many research fields, such as genetic diversity, DNA fingerprint, genetic linkage map, QTL mapping and allele mining (Zietkiewicz et al., 1994; Jun et al., 2008; Zhao et al., 2010; Soren et al., 2015). However, limited SSR markers are available for this orphan grasspea crop compared with other crops (Sun et al., 2012; Lioi and Galasso, 2013; Almeida et al., 2014; Yang et al., 2014). In this study, we implemented RNA-Seq with two different accessions, and RNAs from mixed root, stem and leaf tissues were sequenced. We identified 5,916 SSR markers from the resulting sequence data, designed primer pairs and validated 284 of these markers. Our results showed that 87 (30.6%) of the SSRs were polymorphic and 88 (31.0%) were monomorphic. The rest of the identified SSRs either had no target bands or were too complex to be recognized.

### KASP markers are new and powerful

KASP is a new and powerful tool for SNP testing. Although many SNP testing methods, such as Allele-Specific PCR, Taqman Assay, Molecular Beacons and Microarray-Based SNP Genotyping, are available, they are very expensive (Singh and Singh, 2015). KASP is a new way of SNP genotyping assay based on Allele-Specific PCR with two different forward primers and a reverse primer (Graves et al., 2016). This assay is not only accurate and highly efficient, but is also inexpensive (Khera et al., 2013; Lister et al., 2013). In this study, we used KASP technology to validate 50 SNP primers among 43 grasspea genotypes. The results showed that the array of the 40 SNPs was successfully tested with polymorphism. Two SNPs were monomorphic, and eight markers failed detection. The PIC mean value was 0.2457, which was less than that of the SSR markers, because SNP markers have only two types of alleles, contrary to SSR markers. However, SNPs are very important and widely used because of their stability, dependability and high-throughput. They are highly efficient and accurate for gene discovery (Klepadlo et al., 2017).

## Conclusion

RNA-Seq was performed with two different grasspea accessions with thrice replications for each accession. Sequencing depth was more than 12 Gb for each sample. Based on the *de novo* assembly of sequencing data, 1,970,104 contigs, 142,053 transcripts and 27,431 unigenes were confirmed. A total of 284 SSR markers were validated, 30.6% markers were polymorphic and 31.0% markers were monomorphic among 43 collected grasspea accessions worldwide. For SNP markers, 146,406 SNP loci were called, 50 SNP markers were tested through the 43 grasspea accessions and 42 SNP loci were successfully transformed into KASP markers. The resulting transcriptome data on grasspea have been uploaded to the National Center for Biotechnology Information (NCBI) database. The newly discovered SSR and SNP markers will be useful for genetic improvement of grasspea through breeding.

## Author contributions

XH, TY, JC, and XZ conceived and designed the experiment. XH, TY, YW, RL, and HZ performed the experiment. TY, XH, RL, YY, GR, and DW analyzed the RNA-Seq data. XH, TY, and RL wrote the manuscript. JH, MB, JC, and XZ revised the manuscript.

### Conflict of interest statement

The authors declare that the research was conducted in the absence of any commercial or financial relationships that could be construed as a potential conflict of interest.

## Abbreviations

β-ODAP: β-N-oxalyl-L-α, β-diaminopropionic acid
BLAST: basic local alignment search tool
eggNOG: evolutionary genealogy of genes: non-supervised orthologous groups
GD: gene diversity
GO: gene ontology
KASP: kompetitive allele-specific PCR
KEGG: kyoto encyclopedia of genes and genome
NCBI: national center for biotechnology information
NGS: next-generation sequencing
PIC: polymorphic information content
ORF: open reading frame
QTL: quantitative trait loci
SGR: stay-green
SRA: sequence read archive
UPGMA: unweighted pair-group method with arithmetic means.
