# Supplementary material for: An RNA Sequencing Transcriptome Analysis of Grasspea (Lathyrus sativus L.) and Development of SSR and KASP Markers
Source: Front Plant Sci. 2017 Oct 31;8:1873. doi: 10.3389/fpls.2017.01873 (PMC5671653; doi:10.3389/fpls.2017.01873)
Supplement: Supplementary file 1 [file Table1.DOCX]

Table S1 43 grasspea accessions

| Test number | Accession number | Origin | Geographic region |
| --- | --- | --- | --- |
| RQ01 | S0000001 | Ningxia, China | Eastern Asia |
| RQ02 | S0000009 | Ningxia, China | Eastern Asia |
| RQ03 | S0000013 | Ningxia, China | Eastern Asia |
| RQ04 | Kelanyadou | Shanxi, China | Eastern Asia |
| RQ05 | S0000055 | Shanxi, China | Eastern Asia |
| RQ06 | K887 | Spain | Southern Europe |
| RQ07 | K893 | Eritrea | Eastern Africa |
| RQ08 | K801 | Turkey | Southern Europe |
| RQ09 | K742 | France | Western Europe |
| RQ10 | K814 | France | Western Europe |
| RQ11 | S0000147 | Nepal | Southern Asia |
| RQ12 | S0000126 | Bangladesh | Southern Asia |
| RQ13 | K835 | Yugoslavia | Southern Europe |
| RQ14 | S0000038 | Armenia | Eastern Europe |
| RQ15 | S59 | Tunis | Northern Africa |
| RQ16 | S66 | Latvia | Northern Europe |
| RQ17 | S103 | Morocco | Northern Africa |
| RQ18 | K1336 | Hungari | Central Europe |
| RQ19 | K1313 | India | Southern Asia |
| RQ20 | K1440 | Portugal | Southern Europe |
| RQ21 | K1472 | Greece | Southern Europe |
| RQ22 | K776 | Spain | Southern Europe |
| *RQ23* | *K748* | *Ethiopia* | *Eastern Africa* |
| RQ24 | K406 | Island Cyprus | Southern Europe |
| RQ25 | K420 | Italy | Southern Europe |
| RQ26 | K584 | Italy | Southern Europe |
| RQ27 | K802 | Tadjikistan | Central Asia |
| RQ28 | K842 | Tadjikistan | Central Asia |
| RQ29 | S0000100 | Yugoslavia | Southern Europe |
| RQ30 | K807 | Island Sardinia | Southern Europe |
| RQ31 | S0000127 | Bangladesh | Southern Asia |
| RQ32 | K714 | Poland | Central Europe |
| RQ33 | K791 | Island Sardinia | Southern Europe |
| RQ34 | K1319 | Ethiopia | Eastern Africa |
| RQ35 | K743 | Ethiopia | Eastern Africa |
| *RQ36* | *K91* | *Czech Republic* | *Central Europe* |
| RQ37 | K1364 | Bulgaria | Southern Europe |
| RQ38 | K59 | Afghanistan | Central Asia |
| RQ39 | K1314 | India | Southern Asia |
| RQ40 | K1473 | Turkey | Southern Europe |
| RQ41 | K713 | Holland | Western Europe |
| RQ42 | K475 | Palestine | Western Asia |
| RQ43 | K48 | Czech Republic | Central Europe |
|  |  |  |  |
| All of 43 accessions were used in EST-SSR and KASP markers validation. RQ23 and RQ36 were used in RNA-seq in this study. The seed samples were obtained from Institute of Crop Germplasm Resources, Shanxi Academy of Agricultural Sciences, Taiyuan, | | | |
|  | | | |
|  |  |  |  |
