# Supplementary material for: An RNA Sequencing Transcriptome Analysis of Grasspea (Lathyrus sativus L.) and Development of SSR and KASP Markers
Source: Front Plant Sci. 2017 Oct 31;8:1873. doi: 10.3389/fpls.2017.01873 (PMC5671653; doi:10.3389/fpls.2017.01873)
Supplement: Supplementary file 3 [file Table3.doc]

Table S3A Characteristics of 87 polymorphic EST-SSR markers developed by RNA-seq in *Lathyrus sativus* L.

| Unigene ID | Primer sequence(5’-3’) | Repeat motif | Ta(℃) | Size（bp） |
| --- | --- | --- | --- | --- |
| c45586_g1_i2 | F:CCACTTCCACCTTTGACCAC R:GGAGATCTGATGCAACCCTT | (CT)8cacaccaactcaaaacacaacacctaaaattttccagcaaaataag(T)12 | 52 | 168 |
| c34887_g1_i1 | F:CGAGAAACAGCCTTTACCGT R:GGTTTTTCGAATCCCCAAAT | (T)11aatttac(T)10 | 52 | 239 |
| c46222_g2_i1 | F:TAACAGAATCCTTCCAGGCG R:GGACAGCTTCAGCAACCTTC | (T)10(TC)7 | 52 | 175 |
| c34911_g1_i1 | F:AACAGACGTTATGTCGGTATATACATT R:TTGATCGATTAAGCACGCAG | (TA)8(A)10 | 52 | 320** |
| c45735_g1_i1 | F:CAAGGATCCGGAAGTTCAAA R:GGAAAATTGTAGCGCGAAAG | (A)10 | 52 | 350** |
| c37339_g1_i3 | F:CCAAGGAAGCAAGGCTTTTT R:TTACAATGGTCAGGCAAGCA | (T)12 | 52 | 262 |
| c34700_g1_i3 | F:CACACCCTCAGGTCCTCAAT R:ATGGCACAAAATTTCCCAAA | (T)10 | 52 | 132 |
| c37493_g1_i1 | F:GGCTTCCAAGAACAAAGCTG R:TTACACCAACACATTTCAATGAC | (T)11 | 52 | 333 |
| c36504_g1_i3 | F:TGGTGTGGACAAGCTTTTTG R:GAGCCTTGATCCCAATGAAC | (T)11 | 52 | 299 |
| c5924_g1_i1 | F:TGTTCTTCTGCTTTTCTTCAGC R:AACCCATAGTCCCCATCCTC | (T)10 | 52 | 303 |
| c30856_g1_i1 | F:TTGTCATGCTTCCTGCTTTG R:CAAGGAGCATATGTCCAACG | (T)12 | 52 | 276 |
| c26001_g1_i1 | F:GCGGTTGAAGGTTGTAAGGA R:CCCAACCCACCAAAATCTAA | (T)10 | 52 | 257 |
| c34810_g1_i1 | F:CTGGGAAAAGAAATCGAACG R:GCATGAGGGTCAAAATCTCAA | (T)11 | 52 | 394 |
| c2536_g1_i1 | F:GGGCCATGGCTCAATAGTTA R:GGGCCATATACTCATCCAGG | (T)11 | 52 | 384 |
| c31994_g1_i1 | F:TGATTTGCATTGGTTGCACT R:GCTCCGTATGTTAAGTCTTTCAA | (T)12 | 52 | 282 |
| c27344_g1_i1 | F:AACTCACAAAACCGCCATTC R:CCATGAAAACTCCTGCTGGT | (T)10 | 52 | 247 |
| c35999_g1_i1 | F:GCGGTGATGGTTGTCTTTTT R:CACGGTATTCCACAAATATGC | (T)11 | 52 | 165 |
| c45717_g1_i1 | F:CACCAAAAACCTCTCAAACCA R:TGAGTGAGAGTGAAATGCGG | (A)14 | 52 | 196 |
| c39740_g1_i1 | F:TATTGCAGCAACGCAATAGG R:ACACGTCGACACCGATAACA | (T)13 | 52 | 309 |
| c47533_g1_i6 | F:GCAGCAACAAGAATCCCAAT R:TCACAGCCAGAACAAATCAGA | (T)10 | 52 | 195 |
| c42976_g1_i1 | F:ATTTTGTTGTCAAATTGTCTTGTTA R:CTAATCACAGATGCGCTCCA | (T)11 | 52 | 110 |
| c37015_g1_i3 | F:TCCGGTGGTAACGTTCTCTT R:CAGCATAAAATGGAAGGGGA | (T)10 | 52 | 103 |
| c41936_g3_i2 | F:TGGTCAAACTTTCAATGGCA R:TAAAAACATAGCTGCGGGCT | (T)10 | 52 | 325 |
| c36560_g1_i1 | F:AAATTGCTTTGTTGGCATCC R:ATAGCAGAAGCTCCCAAGCA | (A)10 | 52 | 239 |
| c39067_g2_i1 | F:CCAAAGTCCCTTTGCATTGT R:GCCTTCTAAAGCCTTTGCCT | (T)10 | 52 | 364 |
| c34633_g1_i1 | F:CTGGCACCATAGGGTCAGTT R:CGCGCATACATACAAAGCAG | (T)10 | 52 | 291 |
| c47264_g1_i3 | F:TGTTTTGGAAGGGAGGAATG R:TGTACACTTTACAATCAAAATCCAAA | (T)10 | 52 | 400 |
| c26949_g1_i1 | F:GGAAGACGAAGGGACATGAA R:ACACAAGAAAAAGGAAGTACGAGT | (T)14 | 52 | 267 |
| c46827_g2_i2 | F:TTTAAATTGAAAACAATCTCTCTCC R:TGAAGAAAGTTGCATGACGG | (A)13 | 52 | 118 |
| c31939_g2_i1 | F:CATGATGGGGACTTAAACGG R:TTGCGGTCTCTTCCCTTCTA | (A)10 | 52 | 333 |
| c39234_g1_i1 | F:GTGGATTTGCTTTGGGATGT R:TTCTTGACCCATCACGTTTG | (T)12 | 52 | 352 |
| c36524_g2_i1 | F:GTGTTCCCAGCATTGCTTTT R:TATCCACAATTGGCTCCCTC | (A)10 | 52 | 133 |
| c36087_g1_i3 | F:GGTGCATTCTGGTTTTCCTC R:TGATCCTGGTAAGTCAGCGA | (T)10 | 52 | 328 |
| c39249_g1_i4 | F:ATCCGGGTTTTCAATTTTCC R:CATAACTGCTTTTGGGGCAT | (T)11 | 52 | 163 |
| c47146_g1_i2 | F:TATTTTGCTCAAAGACGGGG R:ACACAGGTCGTTCTCCACAA | (A)14 | 52 | 193 |
| c46770_g1_i1 | F:ATCCTCCACAAGGATACCCC R:TCATTTTATTTTGGTTTGAGCTT | (A)10 | 52 | 323 |
| c46049_g2_i1 | F:TGTCTTTACCGGCCTCTGTT R:CTACCCTACAAGCCTGCTGC | (TG)6 | 52 | 221 |
| c47694_g1_i2 | F:TTCTGAAGATTGTTGCTGCG R:CGTTCGTCTGGAGTTCCACT | (TG)6 | 52 | 102 |
| c34957_g2_i1 | F:TCCCTGTATTCATTTTGTTTTCA R:TTCCATTGATGATGAGGGGT | (CT)8 | 52 | 159 |
| c34100_g1_i1 | F:CCCTTCAAACTTCAAAACCAA R:AGGAAGGAAAGTTGGTCGGT | (CT)8 | 52 | 200 |
| c46949_g2_i1 | F:TCCGTAGCGAATCAAGTGTT R:TTGGCGCATATGTTGGAGTA | (AG)7 | 52 | 172 |
| c75340_g1_i1 | F:GGAACACACAAGAAAAATCTGTCA R:TAGAAACTTGCACGCACCAC | (AG)8 | 52 | 125 |
| c46079_g1_i6 | F:TTTCTTTTTCATTTTTCTCCTTAAA R:TGCAATAATTTGGGGAAAGG | (AT)6 | 52 | 224 |
| c47441_g1_i7 | F:AGAAACGCAGCTTGTCCACT R:CGGTGTGTGAGTGTTTTTGC | (TC)7 | 52 | 101 |
| c40819_g1_i1 | F:TGATTTCAACGACTCTCCGA R:CACTCATCAATTTAGCCGCA | (AT)8 | 52 | 348 |
| c38694_g1_i1 | F:CGTCGGTGACTAGGGAGAAC R:AGAGTTGCCGGAGAGTGAAA | (CT)6 | 52 | 223 |
| c37675_g1_i1 | F:GGATTTTAATCAGCTTTTGCG R:TTTCCTCCTGCCATTGTTTC | (CT)6 | 52 | 430** |
| c43047_g1_i1 | F:TGTGCCCATTCAACAAACAT R:CGAGAAGAACGAGAAGTGGG | (CT)7 | 52 | 227 |
| c37441_g1_i2 | F:ACAGGTTTCCGAAGCATACG R:CAAGTTCAAACTTCGACGCA | (TGG)5 | 52 | 233 |
| c45302_g2_i2 | F:TCTTCAACCCAATCCATCATT R:ATGAAGTTTTGCGACTTGGC | (CTA)7 | 52 | 101 |
| c38894_g1_i1 | F:GGGTTTGAGGAGTTTGGACA R:TCCTCTTCATCTTGCGGTCT | (GAT)6 | 52 | 224 |
| c38070_g1_i2 | F:GAGAAAAATAACCACCGCCA R:CACACAGCAACACGTCCTCT | (ATA)5 | 52 | 209 |
| c31592_g1_i2 | F:TTCGTGTGCAAAACGTTCAT R:GATTTCCTGATTGCTCCCAA | (CTA)7 | 52 | 195 |
| c40146_g2_i1 | F:GGATGAGCACAGGAACCCTA R:CCCTGACTGCACCCTATGTT | (AAC)6 | 52 | 154 |
| c47638_g1_i3 | F:GGTGCAGTGCTTGAAGATGA R:TTAATGTCCGACGAAACGAA | (TGG)5 | 52 | 160 |
| c41017_g1_i2 | F:ACATTATGCGCAACGGTGTA R:TCAACCTTTCATCTCCGACC | (GAG)5 | 52 | 124 |
| c36717_g1_i1 | F:CACCAAAAGAGAAGGACAAGG R:GGTTGATTAGCCTTAGGGGG | (AAG)5 | 52 | 207 |
| c33181_g1_i1 | F:ATGGCTGAGGAGCTTTTTGA R:TCACTTCCGGAATTCTCACC | (GGA)5 | 52 | 187 |
| c37943_g1_i4 | F:ATGGAATGTTGGGGAAACAA R:GAATGATGCACCAATTGCAC | (ATG)5 | 52 | 185 |
| c61294_g1_i1 | F:CTTGGCCAGGAATTGAGTGT R:GCAGAAGGCTTACAAGGTGG | (TGA)6 | 52 | 130 |
| c44073_g1_i3 | F:CGACAGTTGCGACCAGTCTA R:GATTCGGGATTTTTGGGTTT | (TTC)6 | 52 | 271 |
| c35660_g1_i6 | F:TTTTGCCTTAATCTGCACCC R:TTCCCAACACTTTTGCATGA | (TCT)7 | 52 | 209 |
| c46699_g1_i2 | F:GTGCCAGGTAAGGGAACAAA R:CAAGTGGATTTGCATGTTGC | (GAT)7 | 52 | 187 |
| c30552_g1_i2 | F:TCATTGGTCATTATATGAGGCAG R:AGAAAGGGAAGAAAAGGGCA | (CAAA)6 | 52 | 100 |
| c43025_g1_i3 | F:GTTTTGGGGTTTCCCATTTT R:CACATCCAAACCTTTCAGCA | (TGTT)5 | 52 | 245 |
| c39130_g2_i1 | F:TTTGACGATGAATGGGATGA R:AATTTGCGCGGTTAAACAAC | (GAAGAG)10 | 52 | 264 |
| c43981_g1_i2 | F:GGGGGAGGATGTTGACTTCT R:CATACCATCGCATGTGGAAG | (T)11 | 52 | 338 |
| c45148_g1_i1 | F:AGGATGTTGACATGAAGGGC R:TCGAAAAACAGCAACAATGC | (T)15 | 52 | 321 |
| c43652_g1_i5 | F:TGTTGTTGGGAATTTCGTGA R:CCAAGGCGTGAGCTATCTTC | (T)10 | 52 | 362 |
| c13442_g1_i1 | F:GTTTACACCGGAAGCTTGGA R:CACGCCCAATAGTCCATTTT | (T)10 | 52 | 365 |
| c35336_g1_i1 | F:ACACCCCACTCTCTCTCCCT R:TTGAATGAGGCTCTCGGAGT | (T)10 | 52 | 350 |
| c41895_g1_i1 | F:ATTTTTCAACGGATTGCAGG R:TCGCAAGTGCACAACACATA | (T)10 | 52 | 247 |
| c43144_g1_i1 | F:CCTGGTATGGCTATTGAGGC R:CCCGATTTTGATGTTTTACACC | (GTT)5 | 52 | 338 |
| c39279_g1_i12 | F:TATGCACTGATCCAATCCCA R:TTCCCCTTTGTTCCACACAT | (ATC)6 | 52 | 276 |
| c31412_g1_i1 | F:TTGCAGAATCCAAAGCAGAA R:CCAAAAGACCAGCTGACACA | (AAC)7 | 52 | 327 |
| c35761_g1_i2 | F:ATGTAGGCGTTACTGGACGC R:AATCTCCGATTTGAAACCCC | (ACT)5 | 52 | 302 |
| c34069_g1_i1 | F:CCTTTTCATTCCTTTCACGC R:GCTCTGTTGTTGTTTTGCGA | (ATT)5 | 52 | 285 |
| c36754_g1_i1 | F:TTCCATGGGTTCCACATTTT R:ATTCATTCCGTGTGGAGGAG | (CCT)5 | 52 | 373 |
| c41774_g1_i5 | F:TCTTCAACGCTCCACAACAC R:TGAGGGAGGTTGAAAAATGG | (AAC)6 | 52 | 346 |
| c35787_g1_i1 | F:GAGGTGCAACTCATTCTGCAT R:GCTGTACTGTCTGATGGCGA | (T)13 | 52 | 180** |
| c46898_g1_i2 | F:TTGACACAGGGTTTTTGTGAA R:AGGTCAACCTTGTCCAAACG | (T)11 | 52 | 342 |
| c43223_g1_i7 | F:AAACTACCAAAAACGTTCCACA R:TGGAGACGATGATGAATGGA | (CAT)5 | 52 | 390** |
| c43785_g2_i4 | F:TTTGAAGGCTACGCAGGAAT R:CAGAACCAATCCCTGCAACT | (TGG)5 | 52 | 490** |
| c12163_g1_i1 | F:AACCTGCAGGACCACTCAAC R:CCAGTTTCCTCTCGAAGCAC | (GAT)6 | 52 | 310** |
| c32136_g1_i5 | F:TTCCTTCTTTCCTTTCCGCT R:CCTCTTAGGAATTCGCATCG | (TTA)6 | 52 | 378 |
| c45378_g1_i1 | F:CACCACATCCACACACACCT R:CCAGAGTTGTGAAAGTGCGA | (ACACA)5 | 52 | 316 |
| c35626_g1_i2 | F:GGGCTTTGTTTGATGTGGTT R:TACTCGGTGCCTGTTTACCC | (ATGAT)5 | 52 | 277 |

*Note*: *T*_a_ = annealing temperature；* means polymorphic and the other means monomorphic；** mean that the predicted value is unequal with the actual value and the actual value is listed.

Table S3B Characteristics of 88 monomorphic EST-SSR markers developed by RNA-seq in *Lathyrus sativus* L.

| Unigene ID | Primer sequence(5’-3’) | Repeat motif | Ta(℃) | Size（bp） |
| --- | --- | --- | --- | --- |
| c64312_g1_i1 | F:CCTTCAAAATCTCCCCCAAT R:TTGCATTATTGGTTGTTGCC | (TCA)5ttatcgttttcatt(ACA)5 | 52 | 230 |
| c45553_g2_i2 | F:GATTCTTCAGCTGCCGTGTT R:TCCATCTCCGTCGAATTCTT | (T)11(TTG)5 | 52 | 397 |
| c42521_g1_i2 | F:CCAAGGGAAGAACATTGGAA R:TGTAGTTTCCCACAGCAGCTT | (T)13 | 52 | 217 |
| c29318_g1_i1 | F:TTTGGAGAGATATGCAGGGG R:CAGCGGCTCAAATACACAGA | (A)10 | 52 | 135 |
| c32350_g1_i2 | F:AGGCCATGATGGACAGAAAC R:TGTTCCCCACAATCTGAACA | (CTT)5c(TGG)5 | 52 | 190 |
| c35712_g1_i1 | F:TATGTGGGGGCATACCAACT R:TGAAAATGCCAAAACAACCA | (T)10 | 52 | 320** |
| c35039_g1_i2 | F:CCTTGGAATGAGAGGCAGAG R:GTGGTGAGGGAGAGGATGAA | (T)12 | 52 | 204 |
| c38468_g1_i2 | F:CTTTCAGCTTGGAGAAACGG R:TCACATAACTGCATTACCTCTCAG | (T)12 | 52 | 244 |
| c14670_g1_i1 | F:AGATGGAGGTGGTGTTGGTC R:AGCATTGAATGATGTGCCAG | (T)10 | 52 | 262 |
| c36638_g1_i1 | F:TCATGTCAATATCCGCTCCA R:CAGTGTCCATCACGCTAGGA | (T)10 | 52 | 460** |
| c40928_g3_i1 | F:TTTCTTAATCACCATCAACTAATCA R:AAACACGGCACCGACATATT | (T)12 | 52 | 123 |
| c37825_g1_i11 | F:TTTATCTCAAGCTGGGTGGG R:ATCAAGAACCAATGCCGTTC | (T)11 | 52 | 330 |
| c42815_g1_i2 | F:CCAACACAAGTACACAATCAAACA R:GTTAACAGGCTTCAAACCGC | (A)10 | 52 | 340 |
| c32062_g1_i1 | F:GGTGGATGTGAGGTTCAAGG R:GCTCCATTACAAGTAAAATTCAGAAA | (T)11 | 52 | 268 |
| c39144_g1_i2 | F:AATTTCCCTCATTCCCATCC R:CGATTAATCCAACGGTTGCT | (A)12 | 52 | 238 |
| c47941_g1_i1 | F:AGTGATTCAAATGGCCAAGC R:ATGCGTTATCGTCGTCTTCC | (A)11 | 52 | 170** |
| c45707_g2_i2 | F:TGGTGGGAGATTTGTGTTGA R:TTCTAGCAGGCGGCATAAGT | (T)10 | 52 | 344 |
| c45053_g1_i1 | F:TTCAAACTACCCCGCAAAAC R:GCTGTCACTCGAAGTCTCCC | (A)13 | 52 | 490** |
| c24824_g1_i2 | F:AGACGCAATAAGTCATGGGG R:TCCATGTTTCAAATTCAAATGC | (T)10 | 52 | 112 |
| c45182_g1_i1 | F:GCTGTTGTTAAACCCGGAAA R:ACGATGAATTTGGCACTGCT | (T)11 | 52 | 393 |
| c31692_g1_i1 | F:CCAAGTTGTTCCAGTTGGGT R:AAGCATTAAACCCATGCACA | (A)10 | 52 | 232 |
| c34915_g1_i2 | F:CCCGTTCAGTAGAATTCCGA R:GTGAGCGGTTCCAATTTGTT | (A)11 | 52 | 249 |
| c41686_g1_i1 | F:CGAAAGAGAAGGTTTCGCAG R:AAACAGGTGCCCAAAAATAAAA | (T)10 | 52 | 265 |
| c32495_g1_i1 | F:TTTTTGCACAAGAATCCCAA R:TGTCAGCCTTTCGGTATGAA | (A)10 | 52 | 346 |
| c47166_g1_i4 | F:GAATGAGTGGCTGAGTGGGT R:GTGCCTCTCCACAATCTGGT | (T)13 | 52 | 341 |
| c43753_g1_i2 | F:CATTTGGGGAAAAACTCCAA R:AGTCACCACGGCATCTCTTT | (A)10 | 52 | 260 |
| c35392_g1_i2 | F:ATTTGCGGAGCGAGAGTCTA R:CCGGTGATAGCGTTTGATTT | (A)14 | 52 | 193 |
| c44432_g3_i1 | F:AGCATCTGCTGGATCGAGTT R:TTCCCGCAGACAAAATAACC | (T)10 | 52 | 323 |
| c26186_g1_i1 | F:AGCCCGTGAACACTTCTCTC R:GTTTTTGCGTCTCAATGGGT | (A)10 | 52 | 279 |
| c41340_g1_i1 | F:GCCACTCTTTCTCTAACCTCCA R:GGGAGAAATGTTGGGGATTT | (A)10 | 52 | 223 |
| c46515_g1_i2 | F:GCTCCAAGTGTGGCATAACC R:CTCCTCAACCTGCTTGCTTC | (T)13 | 52 | 110** |
| c41674_g1_i2 | F:GGGTTTGAAGGATCGACAGA R:GGATTGATGAGACTGCGTGA | (T)10 | 52 | 261 |
| c43095_g1_i4 | F:TGCGTTGGGTTCAAATTACA R:TTCAACGTTATCAATGGGCA | (T)10 | 52 | 251 |
| c41006_g1_i1 | F:GTGTTTTTCCCGCATGACTT R:GCAGGGTAGGCAAAACCATA | (T)10 | 52 | 364 |
| c44884_g1_i1 | F:CGCTCTTCTTTCCCATTCAA R:ACAAAGGTGGACGATGGAAG | (A)10 | 52 | 193 |
| c54572_g1_i1 | F:AAGATCACGTGGTTGAAGGC R:ATTTTGCCACAATGCTCACA | (A)11 | 52 | 124 |
| c32288_g1_i2 | F:TGATGAGCTTGGTGGTTTTG R:TGATCCTTCCTTTTGCCATC | (T)12 | 52 | 151 |
| c46117_g1_i6 | F:TGTACCAGCAGCAGGAACTG R:TTATTTTGGATTCCTCGCCC | (A)10 | 52 | 367 |
| c18532_g1_i1 | F:TTTTCAACAAAATTGACATCCA R:CCCAAATATTTTCCGTTCCA | (T)11 | 52 | 144 |
| c28118_g1_i1 | F:CACCACCAACAACAAAACAGA R:GGCATTTGAATTCCCTTTGA | (A)10 | 52 | 130** |
| c41549_g1_i1 | F:GCTGCCCAAGCAAGTTCTAC R:GGCAGTAAACCAGTGGAAAAA | (T)13 | 52 | 316 |
| c26052_g1_i3 | F:ACAGTGGCTAGGGGTTGTTG R:GCAGCAGTTATTGCACAGGA | (T)10 | 52 | 185 |
| c43616_g7_i1 | F:TGCTGCTTCATCCCTCTCTT R:ATGATGGAAACGTTGAAGCC | (CT)10 | 52 | 233 |
| c39204_g1_i1 | F:ATATGCCTTTTGCATGGACC R:CCCTTTACGCAAATTTTGGA | (CT)6 | 52 | 270** |
| c33658_g1_i3 | F:TGCATGTAACCAACTCCACTT R:ATGGCAGACATCCTTTCACC | (TTG)6 | 52 | 392 |
| c47334_g2_i1 | F:GTGAAGAAGGTGGTGGCATT R:CCTTCTTCGACGACCACTTC | (GAT)7 | 52 | 230 |
| c39015_g1_i1 | F:AGAGGATCTCCTCCACTCGC R:TCCCTCTGATAGCCAAATCG | (ATT)5 | 52 | 330** |
| c45759_g1_i1 | F:ACCACCACCGTGATTGGTAT R:GGGAAGCGTTATGAGTTGGA | (GCA)5 | 52 | 321 |
| c41553_g2_i2 | F:AACTGCAAATGGAACTTGGG R:AAGAGCGATTCGACGATGTT | (TGG)5 | 52 | 136 |
| c36455_g1_i4 | F:CCTTGCATCATGTTGTTGCT R:AAAAGAGCCACTCGTTTTCG | (ATT)6 | 52 | 145 |
| c35576_g2_i1 | F:TTTGTGGTGAAACATGGTGG R:GGCTTCGGAGAATCAAGAAA | (TCA)5 | 52 | 199 |
| c47029_g1_i1 | F:TGCAGTTGCTAGGTATCCCC R:ATCCACGCCATCATCTTCAT | (GAT)5 | 52 | 289 |
| c45350_g2_i1 | F:AGTTTTCAACCCCAACACCA R:GGGACCACTTTCCGGTTAAT | (ACA)5 | 52 | 301 |
| c63814_g1_i1 | F:TAAGTCCGGTAGGTTGGTGC R:AAAAGAGTGCGAAAGCGAAA | (ATC)5 | 52 | 327 |
| c47420_g1_i8 | F:AGATGTGGGTTATCCGGTGA R:GCAAGAATGAAAGAAACAATGC | (TAT)5 | 52 | 261 |
| c39923_g1_i9 | F:CAACCACTCACCCAAAACCT R:GTGCCGTGGAAAAGTGAGAT | (TTC)5 | 52 | 316 |
| c41960_g1_i3 | F:GAAAATTGTGGTGGTGGTCC R:GACTTGGGCCTGAAAAGTTG | (CAA)5 | 52 | 330** |
| c31176_g1_i1 | F:AAAGCCTCCCTCCACTCTTC R:GAAACGATAAGGGGGTCCTC | (ACA)5 | 52 | 280 |
| c6540_g1_i1 | F:AGGAATGGTTTGGTGGAATG R:CGGCCATGGCATACACTACT | (TGA)5 | 52 | 222 |
| c33353_g1_i3 | F:TCTCGCAATTTCCAAGAACC R:GAGTGCCACTGGTTTCCAAT | (AAT)5 | 52 | 221 |
| c46044_g3_i1 | F:TTTTCTCAAAGCTGCTGGGT R:CCATATGTACCACCACAACCA | (TAG)5 | 52 | 255 |
| c47803_g1_i3 | F:TCTAAGGTTGGAAACGGTGG R:TCTCTTTTCGTCGCGAACTT | (AGG)5 | 52 | 373 |
| c47011_g1_i4 | F:TTTTCGGATTCGTCTTTGGT R:TACCAACCCTACCCAATCCA | (TTG)5 | 52 | 313 |
| c45300_g3_i1 | F:CAGGTTGTCGTGGGAAGTTT R:ATCGGCAGATGAATTGTTCC | (AAC)7 | 52 | 261 |
| c57411_g1_i1 | F:CATGCCTGAATGTCCAGAGA R:GGTTCCTGGTGGACCTAACA | (ATC)5 | 52 | 90** |
| c64692_g1_i1 | F:GCCAACCATGGAAGCATAAC R:AACCAAATCCATGGGATCAA | (GTT)5 | 52 | 102 |
| c44550_g1_i1 | F:GTGGCATGGGTTGCTTATCT R:ATGTCTCGCGGATGGTTTAG | (GAC)5 | 52 | 220** |
| c10513_g1_i1 | F:GGAAGCAGAACAAACCCTCA R:AGAGTGCCGTTAGGGGATTT | (TCT)5 | 52 | 361 |
| c40150_g1_i4 | F:TGAGTCCGAACTGAGCAATG R:AACGAATTGCCATCTCAACC | (AAC)5 | 52 | 275 |
| c47524_g1_i2 | F:TACCGTACCGTTCCACTTCC R:GCATAGGACGAAAGCGAGAC | (CTC)5 | 52 | 328 |
| c32541_g1_i1 | F:TGAAATTCCAAAAGAAGCCAA R:TGAGGTATTGGAAGTGGAGGA | (CAT)5 | 52 | 342 |
| c31081_g1_i2 | F:TCAACAGCAGTTGGCTATGC R:TCCTGGAATTGGGTAGCTTG | (GCT)7 | 52 | 172 |
| c38789_g1_i1 | F:CTCCCATCCTCCTTCTTTCC R:TTCTGCGACATTGACAAAGC | (GCG)5 | 52 | 264 |
| c9172_g1_i1 | F:TCCTCTTAGAATGGGCATGG R:CCCTCTCATTTTACAACTATGCAA | (TGA)5 | 52 | 210** |
| c32515_g1_i4 | F:CGTTACCATGCCTAGGGTTC R:ATGAGAAGGAGGGAAGGCAT | (CTC)6 | 52 | 154 |
| c32078_g1_i1 | F:GGATGTTGATGGAATGCTGA R:CAGCAGCTGTGTCAAAATCAA | (TTGGA)5 | 52 | 342 |
| c39421_g1_i1 | F:CATTCCAGATAAACCCTGCC R:CATCGCTATCCAATGCAGAA | (T)10 | 52 | 270** |
| c31713_g1_i2 | F:ATTTCAGGTTATCCGGTCCC R:GCCATAAATATGAAATCAGAGGAGA | (T)11 | 52 | 610** |
| c40969_g1_i3 | F:GTGTGTCTTGTGCCATCCAC R:CCCCATTTTCTCCAAAGACA | (T)12 | 52 | 387 |
| c44381_g2_i1 | F:TGGTGCCAAACTGTCAACAT R:GTTTTCCCCATGGGTTTCTT | (T)15 | 52 | 600** |
| c36305_g1_i1 | F:AGCTGGCAGCACATACAAAA R:TCCCTCTCTCATCGCTTGAT | (T)10 | 52 | 355 |
| c43599_g1_i6 | F:AAACGGCTATCACCCAGATG R:GAGTTTCCTTGCAGACCAGC | (A)12 | 52 | 310 |
| c28274_g1_i2 | F:GAGGTGAGGCTTGTTTCTGG R:TCATGACCCTTCACCAAACA | (T)14 | 52 | 386 |
| c44390_g1_i3 | F:CGAAACGGAGAAGGAAATCA R:ACACAATCCAACGACCGAAT | (T)10 | 52 | 336 |
| c30691_g1_i1 | F:GAACATTATTTTCTGAATTTTCATGG R:CAGCCAATTCTTCCTTTGGA | (T)12 | 52 | 140** |
| c31818_g1_i1 | F:AAAGGCTGGCACCTCAAATA R:GGCATCAAAGTAAGTCAGGCT | (TAA)5 | 52 | 380** |
| c33865_g1_i1 | F:ACAAAACACCGAGCAAAAGC R:GTCACTGGGCCAAGGAAATA | (TAT)6 | 52 | 313 |
| c38983_g1_i1 | F:GGTAATAGGTGGGAGTGGCA R:AAAAGTACCATTTTCACCACCA | (CTTTT)5 | 52 | 210** |

*Note*: *T*_a_ = annealing temperature；*means polymorphic and the other means monomorphic；** mean that the predicted value is unequal with the actual value and the actual value is listed.

Table S3C Characteristics of EST-SSR markers with 29 no amplified and 80 too complicated to read developed by RNA-seq in *Lathyrus sativus* L.

| Unigene ID | Primer sequence(5’-3’) | Repeat motif | T_a_(℃) | Size（bp） |
| --- | --- | --- | --- | --- |
| c36242_g1_i3 | F:CGTCACAATGACTATTTCCCC R:CATGGACATGCAAAGAGTGG | (T)10 | 52 | b |
| c43475_g4_i2 | F:CCTCCATTTCACTCATCTACCTC R:TGACGGAGAAAGTTTCGCTT | (TCT)5tcacagttccaacttcagacaaaccaaaatctccttcttcacttctcacacatttccacagatccacaaccatctttctctctccaacacttcat(TC)8 | 52 | b |
| c41414_g1_i1 | F:CACGCGTGTCTTGACTTCAT R:GCTTGGCGGAGTTAGAGATG | (T)10 | 52 | b |
| c47672_g1_i5 | F:CCAAAAACAAAAACAACACAAAA R:AATTCAAGTGCAAGTGCGTG | (A)12 | 52 | b |
| c45737_g4_i4 | F:GCTCATTGCAAAATTTCCGT R:TTCATATTTCCCGACCTTGC | (A)11 | 52 | b |
| c43066_g1_i1 | F:CGTGGTTTGGTCTTGATTCAT R:TAGCCAAAATTCCGATACGC | (T)15 | 52 | b |
| c44426_g1_i1 | F:CTTGTGTGGCTTGGATTGAA R:ACCCCACCTAAAATCAAGGG | (A)14 | 52 | b |
| c26956_g1_i1 | F:TGAAACGGTGAAACGGTGTA R:TCAAATGACATAGACGAGATCCA | (A)10 | 52 | b |
| c30072_g1_i1 | F:CTGCTGGTAGACGGTTGGTT R:GTAGGTGAACTCGGCGAACT | (T)10 | 52 | b |
| c47164_g2_i3 | F:TCCTCACACACTGTTCAGTCCT R:AAACGCAACATTGGCAGATA | (T)10 | 52 | b |
| c34993_g2_i2 | F:TTCAGTTTGTGCTGGGACTG R:ATCCATCCAAACAAACAGGC | (T)11 | 52 | b |
| c17085_g1_i1 | F:TTTTGTTTGTCTTTGTTGCTGAA R:GGCAAATAGAATCGCCAAAA | (CT)7 | 52 | b |
| c37909_g1_i5 | F:AATGGCAAATGTGGGTTTGT R:GCACTTTGCTTAGATTTGGCA | (CA)6 | 52 | b |
| c24001_g1_i1 | F:ATGTTTTTCAGTGGCGAAGC R:CAAAACAGAGCAAAAATAGCCA | (TA)6 | 52 | b |
| c41862_g2_i1 | F:ATCTTCGTGTTCTTCCCACG R:ATGGCTCTTTTGAGCATCGT | (CT)9 | 52 | b |
| c258_g1_i1 | F:CCAAGGCTCCTCAGAGGTAA R:AGAGGGTTTGCATGGTTTTG | (CT)6 | 52 | b |
| c35923_g1_i1 | F:ACCAACATGGAGATGCAACA R:CCTTCTCCGCTCAAGTTCAC | (CCA)7 | 52 | b |
| c47859_g2_i2 | F:GGCCCTTACCCTCTTTCATC R:CAAAAGGGAGAGACGGACAA | (TCT)5 | 52 | b |
| c38139_g1_i1 | F:GCCATTTTTGTTTTCTTACTTTTC R:GTTACTCCGGTGACAGCCAT | (TTG)5 | 52 | b |
| c25469_g1_i1 | F:TCCCAATCTCAACCAGAACC R:CCAAATCAAGCCACGAGTTT | (ACA)6 | 52 | b |
| c42058_g1_i1 | F:TCAGGAACAGATCCAGGCTT R:AGCATCCTTTGAGCTGCATT | (TTG)5 | 52 | b |
| c34650_g1_i1 | F:CAAGTCCTCTTGGTTTGCGT R:TTTGCCACCAAATCTCCTTC | (GCT)6 | 52 | b |
| c29186_g1_i1 | F:CCCACATACCCATGACAACA R:TTTTGACACTGTCCCCTTCC | (AAC)5 | 52 | b |
| c15079_g1_i1 | F:CCCTATGCAGCAGAAGGTGT R:TTGCAAGGCAACTCACTCAC | (GAA)5 | 52 | b |
| c6160_g1_i1 | F:AACAGAAAGCTGCTGCCATT R:TGATGTTGGTGGCATAGCAT | (TCA)5 | 52 | b |
| c43066_g1_i1 | F:TGTTTCCCACACACTTCCAA R:AAGGGAGGTGCAACAACAAC | (TCT)6 | 52 | b |
| c37246_g2_i5 | F:TGAAGCTTCGCCTTTCATTT R:CGGTAGCAGGTTTTGGTGAT | (TTC)5 | 52 | b |
| c45374_g1_i2 | F:TCATTGTGCTGGGATTTTGA R:TTGATATTCTTTCCCAATCACTCA | (TAT)6 | 52 | b |
| c46386_g1_i5 | F:ATCTTCAAAACCCCAATCCC R:GCAAGAGCTTTTTGTCTGGC | (ATTCCA)5 | 52 | b |
| c37902_g1_i1 | F:TGTCACCGAAGGAAAGAACC R:AAATGCCCTTACACATTGCC | (A)12gaaaaaaactcaaaatcgtgtgtttttctttttttcctatatctttttgggagattcattttgttttttttgttcttttgtgtgagac(T)10 | 52 | c |
| c34296_g1_i1 | F:CAATGGGAAGCTTTGATGGT R:ACAACCAAATCCACTGAGCC | (A)10 | 52 | c |
| c17816_g1_i1 | F:ACAGCAGCAACAACATCAGC R:TGACCCATGTTCTCACCAAA | (CAG)5ccgcaacc(ACA)5 | 52 | c |
| c38417_g1_i2 | F:AGCAAAAGCCTTGCTCCATA R:AGAAAACGGAACCTTCACACA | (T)10ccaagacaaagggtaaacttttgaattcgtgttatccctttattactcgttgaagggca(T)10 | 52 | c |
| c12998_g1_i1 | F:ATCCATGGAAAACCAAACCA R:GCCACAAAATATACAAAACAAGTTCA | (T)10 | 52 | c |
| c45279_g1_i2 | F:CCCTCCCAATTCCATTAACA R:TACCAGCTCCCATGTTGTCA | (A)10 | 52 | c |
| c47128_g1_i3 | F:GGAAAATTCCCGCTTTTCTC R:ATCAAACTTGGAGGCCAGTG | (T)10 | 52 | c |
| c47046_g1_i5 | F:ATTACGCCACCCAATCAAAA R:AAAGGCAAAGAAGGAGGAGG | (T)12 | 52 | c |
| c38525_g1_i2 | F:AAGTCCAACCACCCTAACCC R:GGGAGCAACAACTGAGAAGC | (TCT)6 | 52 | c |
| c35225_g1_i3 | F:TTCCAAAAGCCCAACCTATG R:AGCCAAAGCCTCTGCAACTA | (CAT)5 | 52 | c |
| c33951_g1_i2 | F:GGGTAAAATCGTCTCGTGGA R:CTGAGGTGTCGCCGTAATTT | (CTC)5 | 52 | c |
| c41565_g1_i3 | F:CACTTTCACCATGGCTTCCT R:TCACCGTAACTTCCGAATCC | (TTC)5 | 52 | c |
| c33138_g1_i2 | F:TGGAACTGCATTGTCAAGGA R:TCCACCTTCACCTTTCTGCT | (GAGAAT)5 | 52 | c |
| c40526_g2_i3 | F:CCATTTTAATACACCACCCACA R:GATTTTGGTGGATTTGGAGG | (CT)7ccctcaaagccacacaaagaaaaaaaacaactcaacttgaccaaccttaaagttattactatataaccctaaccacataacatacccaaacccctcttc(A)14 | 52 | c |
| c42693_g1_i3 | F:TGTTTGATTGGCTGACCAGA R:GGATTTGGAAAATTGTTGGG | (T)16accaaaatgcatgatttttttctctctataaatagagacttggttcatttgatttggacacag(A)10 | 52 | c |
| c41235_g1_i2 | F:GCGAACCCCAACTAGTCGTA R:TCCAGCCTTATTCAAATGGG | (T)10 | 52 | c |
| c37340_g1_i1 | F:CTTGTGAAATGGCGAATCCT R:TGAGCAATATTTACATGGGCA | (T)10 | 52 | c |
| c28283_g1_i1 | F:TCATACGTGTGCTCTTCCTTTC R:GATGGGCTGAATCACTTGGT | (T)12 | 52 | c |
| c30093_g1_i1 | F:CATGGATGATGCTTGTTTGG R:TATGCAAACCCTGCTCATTG | (T)10 | 52 | c |
| c47015_g1_i1 | F:TTGCTGCTAACCTTGGCTTT R:GTGCCTCCGTAGTCGTTAGC | (A)10 | 52 | c |
| c44096_g1_i1 | F:CACTAAGAACAAATCAACAACAATCA R:GTGGAACGAAACGGAAGGTA | (A)15 | 52 | c |
| c30610_g1_i2 | F:GGTTGGAATTTGGTATTCAGTTG R:CCTTCATCTTCTGTGCTTCCA | (T)11 | 52 | c |
| c26933_g1_i1 | F:TGAGGCAGTTGGAGGAGAAT R:TCCTGAAACAAACAAAAATAAATCA | (T)11 | 52 | c |
| c35797_g1_i2 | F:AGGTTTGGGTTGGGAGAAAG R:AGGCTTGTACCTTGAGCGAA | (A)10 | 52 | c |
| c41878_g1_i1 | F:AACGCTCAACGAACCAATTC R:TCCAGCAACATTCCAAGTACC | (T)10 | 52 | c |
| c24233_g1_i1 | F:TCCTGAAAATAACAAATAATATTGGAA R:GTCCAACACTTTCGGCAAAC | (A)10 | 52 | c |
| c42206_g2_i8 | F:TGGTGTGATTTTGTGCAGGT R:ATCCAAAACAAACCGCTCAC | (T)13 | 52 | c |
| c47207_g1_i1 | F:TATCGGTATCGACGCCTTTC R:GCATTGACAAAACTGGGCTT | (T)10 | 52 | c |
| c1197_g1_i1 | F:AAGGTTGGCCGATGTGTAAG R:TCAGCACAACCTCAGAATGC | (T)11 | 52 | c |
| c31430_g1_i3 | F:GATGCTCTTCTTGCCAGGTC R:TATCGAATGGTGGTGTGGTG | (T)11 | 52 | c |
| c34114_g1_i1 | F:ATTTTGGGCTCATGTTGGAG R:CCCGTGATCAATCCAATACC | (T)10 | 52 | c |
| c44179_g1_i1 | F:CACGTGGCACAAAACCATAC R:ATCGGAGAAACCAAGGAGGT | (A)10 | 52 | c |
| c40765_g1_i6 | F:CACATAATTGACCCCTCCCA R:ACCCGAGATATAACCCACCC | (A)10 | 52 | c |
| c37931_g1_i1 | F:CGATGCTTCCTCTCTCCATC R:TTGGAGGATGATCTTGGAGG | (A)10 | 52 | c |
| c41866_g1_i2 | F:GCACAGATTCACCACTGGAA R:GTGTCGGTGGTCCATAGCTT | (A)10 | 52 | c |
| c33634_g1_i1 | F:CGCTCCACCGTTCATCTTAT R:GCCCCTCACACAAATGATAG | (TA)7 | 52 | c |
| c46172_g3_i2 | F:ATTAGGGTTGGAGATTGGGG R:GCTTGCGTCTCCCTTTGTAG | (CT)6 | 52 | c |
| c32953_g1_i1 | F:CGAAAAGGCGAAAGAGAATG R:ACGAACATGGTCTCACCACA | (TC)6 | 52 | c |
| c47327_g2_i3 | F:TGGCCGCTAAGTTAAGCTGT R:CTCGAAGACCATTCCTTTGC | (CT)6 | 52 | c |
| c33883_g1_i2 | F:AGGGTGAAAATTCGTGATCG R:CATACACGTGTCGGTGGAAG | (TC)9 | 52 | c |
| c38748_g1_i3 | F:ATTGCGTTGCAGAGTGAGTG R:GGTGCTGCTGCATTTTACCT | (CT)6 | 52 | c |
| c41396_g1_i1 | F:CATACTCATCCACACGCACC R:TGGATCACGGAAAGTCACAA | (CT)7 | 52 | c |
| c46752_g1_i4 | F:CCACAGCCGAAGTCTCTTTC R:GGAGGAAAAGAAGGGGTGAG | (CT)7 | 52 | c |
| c43531_g1_i1 | F:CCCTTGTCTTCCTTTTTCTTCTT R:ATGTATGGATGGTGCGGATT | (TC)6 | 52 | c |
| c47884_g4_i3 | F:TTCTCTCTCAAACCGAATTGTTC R:CGCAGTATCCCACCAAGACT | (TC)7 | 52 | c |
| c38720_g2_i1 | F:TTCACAAAATGCGACCAAAA R:CACGTCTTTTTCAGAGGGGT | (CT)7 | 52 | c |
| c20887_g1_i1 | F:GAATGGTATTTTCTTACGCATTTACA R:GGCTGGATCAATTTCGTCAT | (AT)6 | 52 | c |
| c46920_g1_i5 | F:CCACGCTCACGGGTACTTAT R:CAGGAGCACCAGTCACTTCA | (TC)6 | 52 | c |
| c30812_g1_i1 | F:TCCTCCAGCACCAAAGAAAC R:TTGCATGTTTGAATTCGCAG | (GT)6 | 52 | c |
| c41942_g1_i3 | F:CACCCGGTTACTATGAGCGT R:GCTGACGGCAGAGAAAAGAC | (TC)9 | 52 | c |
| c46541_g1_i3 | F:TTTTCCTCCATACTGCCGTC R:ACAGGTTTGGACCCTGAGTG | (CT)8 | 52 | c |
| c47202_g1_i5 | F:TTTTTCGGGTTTCCGTTTTT R:CTATATTAACAGGCGCGGGA | (TG)6 | 52 | c |
| c32642_g1_i1 | F:GCTCCAACCTTGACCAAAAA R:TCTCTCTCCTTCGTGACCGT | (CA)6 | 52 | c |
| c34035_g1_i1 | F:TTGGGCTGCTGAATTTTTCT R:ACTGCAAAAGCCTCAAAGGA | (GA)6 | 52 | c |
| c41756_g1_i3 | F:ATTTCGTTGCAAAAGCCATC R:TCAGAGTTTGTAGTGGCAATTTTC | (GAT)5 | 52 | c |
| c31571_g1_i3 | F:TTTTTCCAAGTCGCGTTTTT R:GCCCTTTCACAGTTGGATGT | (GCC)5 | 52 | c |
| c39889_g1_i4 | F:CACTCCAACCAACAACATCG R:TCCGGTGCCTCAGATTTAAC | (ACA)5 | 52 | c |
| c43756_g1_i2 | F:GGGAGAGAAGAGGAGGGTGT R:GAATTGATTGGCTTGGGCTA | (TTC)7 | 52 | c |
| c34561_g2_i1 | F:TGACACCAAGGCCTTCAAAT R:TCCACAAAAACCACCAATCA | (TGG)5 | 52 | c |
| c21309_g2_i1 | F:TGGCAGAGAAAAGTTGGCTT R:CCCATGTCATCATCAGATTCC | (GTG)7 | 52 | c |
| c24761_g1_i1 | F:ATTGTTGGTGGTGCCGTATT R:CGTTCTCGTCCATTTTGGTT | (TCT)7 | 52 | c |
| c44201_g2_i1 | F:CTGCTGCTGGAGGAGAAGTT R:AAAGCAGCACCACCATTACC | (GGT)5 | 52 | c |
| c47728_g2_i1 | F:CTATTGCCAAGCGTCATCAA R:GTGCAAGTGTGTCACCCATC | (TGA)5 | 52 | c |
| c42212_g1_i2 | F:GTTGAGAGGAGGCAGAGGTG R:ATCTGAACAGCGGAAGCACT | (CAA)5 | 52 | c |
| c37257_g2_i2 | F:GTGGTGTGTGTTTCAAACCG R:AGAACCAACAAACCCATTGC | (TTC)6 | 52 | c |
| c40252_g1_i2 | F:TTCAACCATGACCAAACCAA R:ATTATGCAGTGGAGGCTGCT | (CTC)6 | 52 | c |
| c47206_g1_i3 | F:CTTCTCTTCTTCACCAGCCG R:AATAACAGTGATGCCCCTCG | (TTC)5 | 52 | c |
| c32475_g2_i1 | F:GGACCACACGGATATATGGC R:TTGTACAGTGAAAAGGCCCC | (CCT)5 | 52 | c |
| c46365_g1_i1 | F:TCCACATCATCATTGCATCA R:AGTGGAGAGAAGAGCGGTGA | (CAA)5 | 52 | c |
| c38883_g1_i4 | F:ACACCTTGTTCTAATGGCGG R:GGCGAGCTTCAATTCTTCAC | (TCC)5 | 52 | c |
| c16970_g1_i2 | F:CTTCTTCCTCCCACAACAGC R:TGAGGATAAGGACCAGGACG | (GCG)5 | 52 | c |
| c41501_g1_i3 | F:ATGGCTTCTTCTCTGTTGCC R:GTGGCATCGATGACTGTGTC | (TTC)5 | 52 | c |
| c72761_g1_i1 | F:TGATCTGCAACAACAGCCTC R:GCTCTCCAAACCCACTACCA | (TCA)5 | 52 | c |
| c39113_g1_i3 | F:TGCATATGCTGATTGGTTGG R:CCCGTGGGAGCAATCTACTA | (CTC)5 | 52 | c |
| c31164_g1_i1 | F:TCCACTAAACCAAGCTCGAAA R:AAGTAGCGCGCAATCTATCA | (TTG)5 | 52 | c |
| c5483_g1_i1 | F:ACAGGGGAGTATGCTTCACG R:AAGGCCAATGCTAACAAGGA | (ATAA)5 | 52 | c |
| c45786_g2_i1 | F:ATCACCGGAATTGCTGAAAG R:GGAAGAAAACAATCGGTGGA | (AAAT)5 | 52 | c |
| c42751_g1_i2 | F:CAAAACATGGCACCAACAAG R:GGGAAAGCAGCTATCATTCG | (TTGG)5 | 52 | c |
| c42177_g1_i1 | F:CTTTTCGTCAGCCATTGGTC R:ATCCCATATAGGCTGGGGAG | (AAAAG)5 | 52 | c |
| c35810_g1_i2 | F:TTAGGTGGGGTTGTGATTCC R:TTGAAGGTGGAGAATGGGAC | (TCCTC)5 | 52 | c |

*Note*: *T*_a_ = annealing temperature；“b” means no amplified bands and “c” means too complicated to read.
